# Cardiac expression of microRNA-7 is associated with adverse cardiac remodeling

**DOI:** 10.1038/s41598-021-00778-6

**Published:** 2021-11-10

**Authors:** Manveen K. Gupta, Anita Sahu, Yu Sun, Maradumane L. Mohan, Avinash Kumar, Ajaykumar Zalavadia, Xi Wang, Elizabeth E. Martelli, Kate Stenson, Conner P. Witherow, Judy Drazba, Srinivasan Dasarathy, Sathyamangla V. Naga Prasad

**Affiliations:** Department of Cardiovascular and Metabolic Sciences, Lerner Research Institute, Cleveland Clinic, 9500 Euclid Avenue, Cleveland, OH 44195 USA

**Keywords:** Biochemistry, Computational biology and bioinformatics, Physiology, Cardiology, Pathogenesis

## Abstract

Although microRNA-7 (miRNA-7) is known to regulate proliferation of cancer cells by targeting Epidermal growth factor receptor (EGFR/ERBB) family, less is known about its role in cardiac physiology. Transgenic (Tg) mouse with cardiomyocyte-specific overexpression of miRNA-7 was generated to determine its role in cardiac physiology and pathology. Echocardiography on the miRNA-7 Tg mice showed cardiac dilation instead of age-associated physiological cardiac hypertrophy observed in non-Tg control mice. Subjecting miRNA-7 Tg mice to transverse aortic constriction (TAC) resulted in cardiac dilation associated with increased fibrosis bypassing the adaptive cardiac hypertrophic response to TAC. miRNA-7 expression in cardiomyocytes resulted in significant loss of ERBB2 expression with no changes in ERBB1 (EGFR). Cardiac proteomics in the miRNA-7 Tg mice showed significant reduction in mitochondrial membrane structural proteins compared to NTg reflecting role of miRNA-7 beyond the regulation of EGFR/ERRB in mediating cardiac dilation. Consistently, electron microscopy showed that miRNA-7 Tg hearts had disorganized rounded mitochondria that was associated with mitochondrial dysfunction. These findings show that expression of miRNA-7 in the cardiomyocytes results in cardiac dilation instead of adaptive hypertrophic response during aging or to TAC providing insights on yet to be understood role of miRNA-7 in cardiac function.

## Introduction

Symptomatic heart failure is still a chronically progressive disease with therapeutic strategies that only delays the outcomes. The heart responds to the increasing demands of the body by undergoing hypertrophic response typically classified as physiological or pathological hypertrophy^1^. Physiological hypertrophy is a reversible response to exercise or pregnancy^2^, while pathological hypertrophy is irreversible typically leading to cardiac dilation and heart failure^2^. Cardiac hypertrophy is also observed with aging and is considered to be a physiological response by the heart to meet the increasing demands of the body. As majority of the cardiomyocytes are terminally differentiated, the heart responds to increasing demands of the body by undergoing an adaptive hypertrophic response. However, increased mechanical load on the hearts for longer periods of time leads to adaptive hypertrophy that becomes maladaptive over time resulting in cardiac dilation. Though studies have focused on determining molecular components involved in the transition from hypertrophy to dilation^3,4^, less is known about these pathways. Understanding these pathways becomes critical as it is known that anti-cancer therapeutics including those that target epidermal growth factor receptor (anti-ERBB agents) family^5,6^ leads to cardiac dilation of the otherwise healthy hearts bypassing the adaptive hypertrophic response^7^.

Epidermal growth factor receptors (EGFRs or ERBBs) mediate cardiac hypertrophic response^8^ and are represented by four members, ERBB 1 through 4 that either homo- or hetero-dimerize to mediate downstream signals^9^. Studies have shown that ERBB1 (EGFR1) and ERBB2 are transactivated by beta-adrenergic receptor (βAR) ^10,11^ leading to beneficial downstream signaling. Activation of ERBB2 by neuregulin (NRG1) is considered to re-initiate cardiomyocyte division^12^, while conditional ERBB2-knockout mice are characterized by cardiac dilation^13^ indicating a key role for ERBB2 in cardiac hypertrophic response. Accordingly, cardiomyocyte-specific overexpression of ERBB2 leads to cardiac hypertrophy without heart failure^13,14^. These observations support the role of ERBB2 in adaptive cardiac hypertrophy and consistently, chemotherapeutic targeting of ERBB2 in patients results in cardiac dilation of the otherwise healthy hearts^15^.

Cardiac remodeling including hypertrophic response and dilation is a complex process, in part mediated by microRNAs (miRNAs) ^16–18^, wherein they mediate these effects by binding to complementary mRNA transcripts^19,20^. Despite miRNAs ability to bind multiple mRNAs, it is now understood that expression of miRNAs regulates a specific primary set of proteins resulting in unique phenotypic outcomes^21–23^. Since miRNA-7 is known to target the ERBB receptor family in cancer cells^23,24^, there is increasing recognition that miRNA-7 could be used as a potential cancer therapeutic^25,26^. Given the evidence that anti-cancer anthracyclines are associated with cardiotoxicity^27^, it becomes critical in this context to determine whether expression of miRNA-7 changes cardiac functional outcomes. Since very little is known about the role of miRNA-7 in the heart, we generated a transgenic (Tg) mice with cardiomyocyte-overexpression of miRNA-7 (miRNA-7 Tg) to assess its role in cardiac function. Cardiomyocyte expression of miRNA-7 results in significant reduction of ERBB2 that is associated with age-based cardiac dilation and accelerated heart failure post-TAC. An observation, reminiscent of the cardiac dilation observed with anti-ERBB2 agents^28^. Recognizing that miRNA-7 could have targets beyond ERRB family, proteomics was performed on the miRNA-7 expressing hearts. Networking analysis of proteins showed reduction of mitochondrial membrane proteins in miRNA-7 Tg mice and correspondingly, electron microscopy showed disorganized and rounded mitochondria indicating mitochondrial dysfunction. These studies provide insights on the underpinnings of cardiac dilation as it is the common exacerbated phenotype of heart failure observed in end-stage human heart patients and in patients undergoing cancer therapeutics^28,29^.

## Results

### Cardiomyocyte-specific overexpression of miRNA-7 leads to cardiac dilation

**Figure 1:**
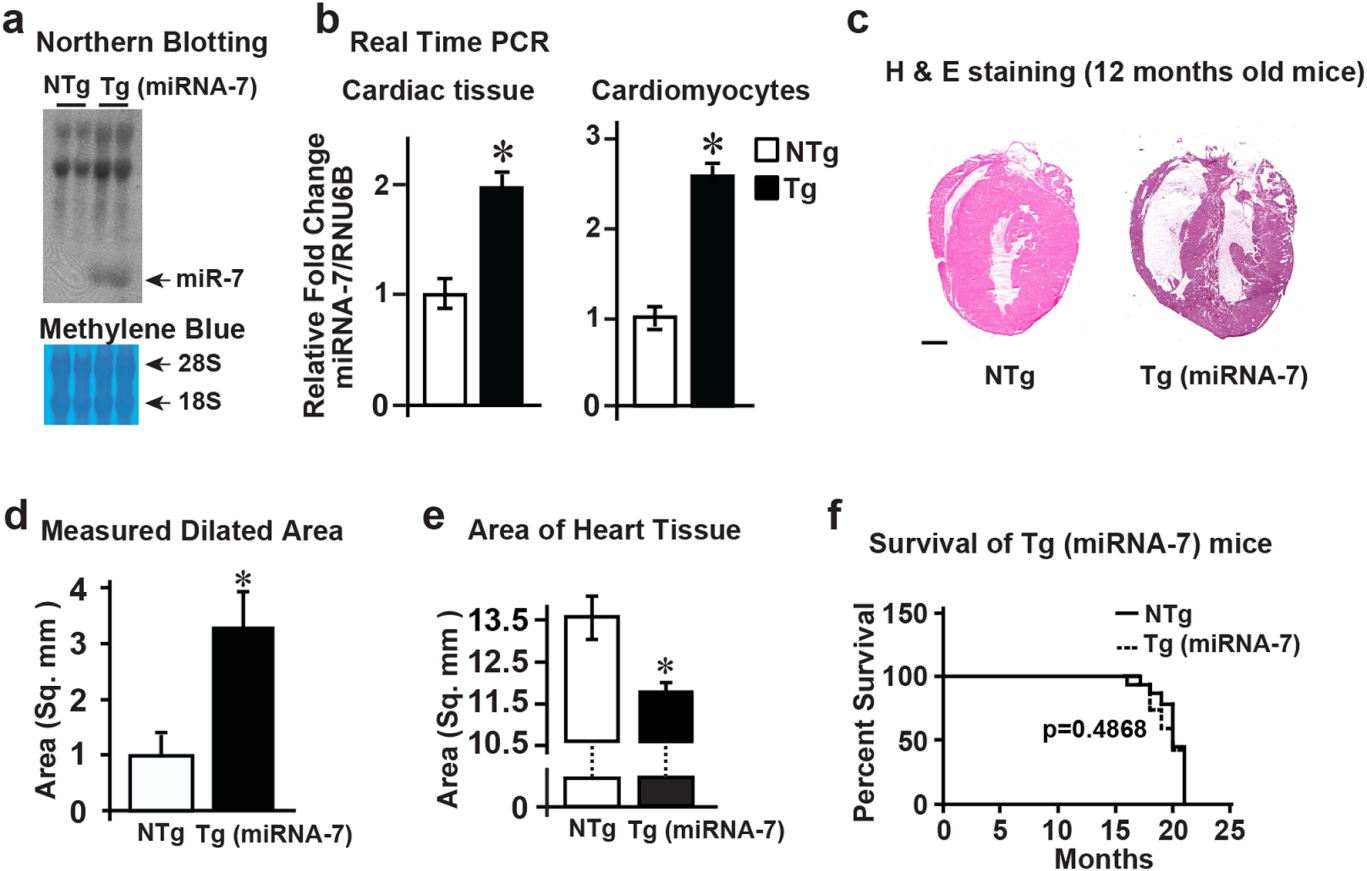
microRNA-7 (miRNA-7) Tg mice have similar life span despite cardiac dilation (**a**) Northern blotting for miRNA-7 in the Tg (miRNA-7) mice and littermate controls (NTg) (upper panel); Methylene blue staining of RNA for equal loading (lower panel) (n = 5). (**b**) Real time PCR analysis for miRNA-7 expression from cardiac tissue (left panel) and isolated adult cardiomyocytes (right panel) from NTg and Tg mice. *p < 001 vs. NTg (n = 5). (**c**) Cardiac sections from 12 month old NTg and Tg mice stained with H & E wherein, Tg hearts are characterized by marked dilation compared to NTg (n = 4). Scale bar 1000 µm. (**d** and **e**) The dilated area (**d**) and the area of cardiac tissue (**e**) was measured with Image Pro Plus using the 12 month old H & E stained heart sections (n = 4). *p < 001 vs. NTg (d) & *p < 0.01 vs. NTg (**e**). (**f**) Kaplan–Meier survival curves for NTg and Tg mice show no appreciable differences in survival between NTg and Tg (n = 18).

To evaluate expression of miRNA-7, northern blotting was performed on mRNA isolated from miRNA-7 Tg and littermate controls (NTg). Marked expression of miRNA-7 in the miRNA-7 Tg hearts was observed compared to NTg controls (Fig. 1a). To evaluate the expression of miRNA-7, quantitative real time PCR (qRT-PCR) was performed on mRNA isolated from total hearts which showed significant expression of miRNA-7 in Tg mice (Fig. 1b, left panel). To further validate the expression of miRNA-7 specifically in the cardiomyocytes, adult cardiomyocytes were isolated from NTg and Tg mice. qRT-PCR was performed on the mRNA isolated from adult cardiomyocytes that showed significantly higher expression of miRNA-7 in the Tg cardiomyocytes compared to the NTg (Fig. 1b, right panel). Since miRNA-7 Tg mice displayed no overt abnormality, H & E staining was performed on heart sections of 12 month old miRNA-7 Tg and NTg mice. Sections from miRNA-7 Tg mice show marked dilation compared to NTg (Fig. 1c), wherein measurement of total heart area using Image Pro-Plus showed increased area in the miRNA-7 Tg mice compared to NTg (Fig. 1d). This was inversely correlated to decreased cardiac tissue area in the Tg mice compared to age-matched NTg (12 months) (see methods) (Fig. 1e). As cardiac dilation is observed in 12 month miRNA-7 Tg mice, survival analysis was performed over the 20 month life span. Despite significant cardiac dilation, Kaplan–Meier survival curves show no appreciable differences in survival rates between Tg and NTg mice (Fig. 1f).

### Increasing cardiac dysfunction with age in miRNA-7 Tg mice

**Figure 2:**
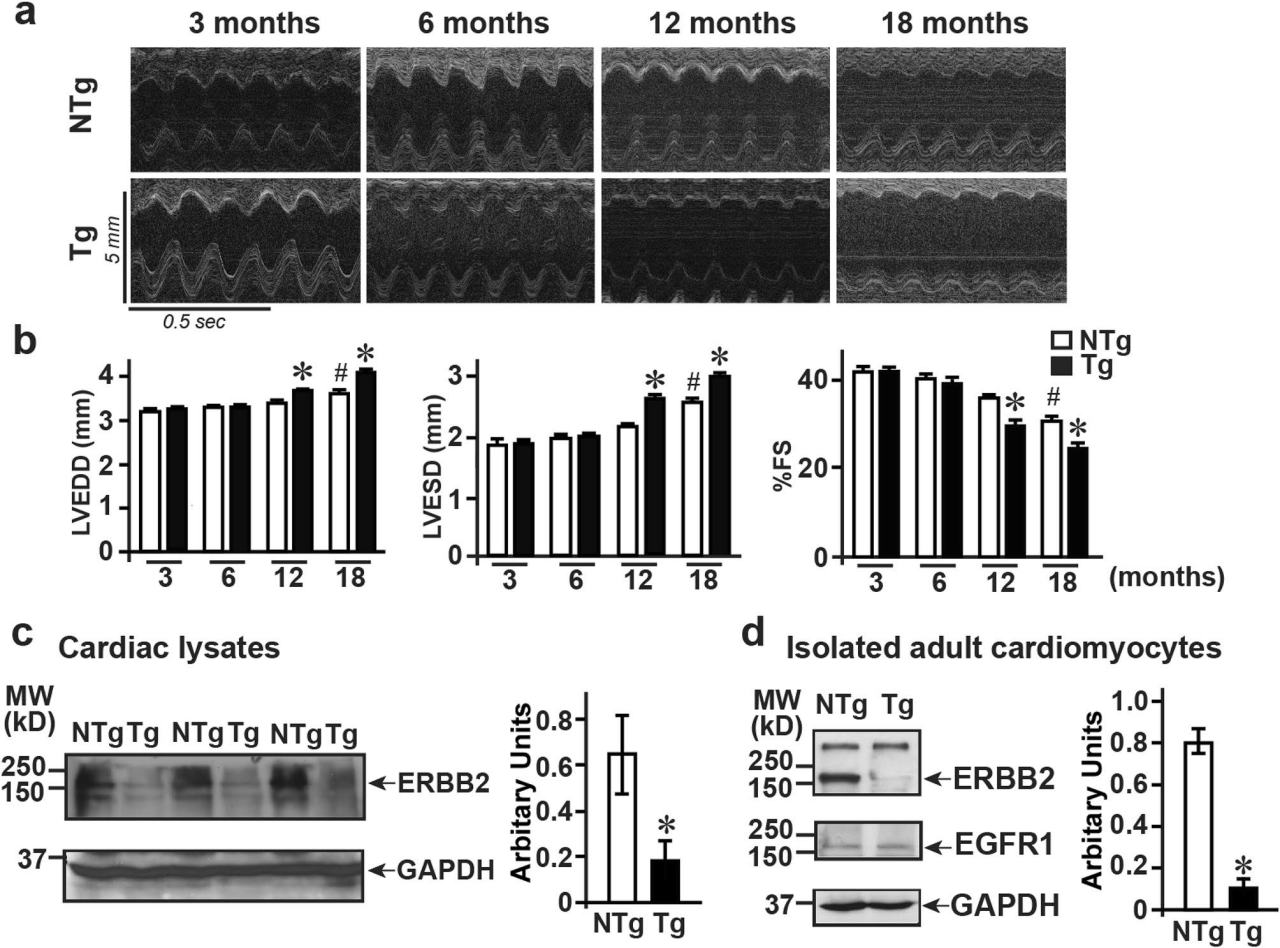
Cardiomyocyte expression of miRNA-7 leads to age-based cardiac dilation (**a**), M-mode echocardiography performed on NTg and Tg mice at 3, 6, 12 and 18 months (n = 12). (**b**), Cardiac functional parameters measured from echocardiography on NTg and Tg mice; Left panel: LVEDD (left ventricular end-diastolic dimension); Middle panel LVESD (left ventricular end-systolic dimension); Right panel: % fractional shortening (%FS) as measure of cardiac function. *p < 0.01 vs. 3 month NTg and Tg; ^#^*p* < 0.05 vs. 3 month NTg and Tg. (**c**) Immunoblotting for ERBB2 in the total cardiac lysates from NTg and Tg mice. Immunoblotting for GAPDH was performed for loading control (n = 8). Summary data represented in the bar graph (right panel). *p < 0.001 vs. NTg. (**d**), Immunoblotting for ERBB2 and EGFR in isolated adult cardiomyocytes from NTg and Tg mice. Immunoblotting for GAPDH was performed as loading control (n = 4–5). Summary data represented in the bar graph (right panel). *p < 0.0005 vs. NTg.

Since there was no difference in the survival between NTg and Tg mice, miRNA-7 levels were assessed with aging in NTg mice. qRT-PCR was performed on mRNA isolated from neonatal and adult cardiomyocytes (3, 6, 12 and 18 months of age). qRT-PCR studies showed that there was no difference in miRNA-7 expression in the neonates, 3 and 6 months old mice (Supplementary Fig. 1a). While, there was significant reduction in miRNA-7 expression in the 12 and 18 months old mice (Supplementary Fig. 1a) suggesting a potential role in age-dependent hypertrophic response. Given reduction of miRNA-7 with aging in NTg mice and similar survival rates despite cardiac dilation in the miRNA-7 Tg mice, cardiac function was investigated in the Tg and NTg mice over 18 month period by echocardiography. M-mode echocardiography showed that Tg mice were characterized by increasing cardiac dysfunction with age as measured by functional parameters (left ventricular end-systolic diameter, LVESD; LV end-diastolic diameter, LVESD; % fractional shortening, % FS) (Fig. 2a, b and Table 1). Significant cardiac dysfunction was observed by 12 months of age that further deteriorated by 18 months (Fig. 2a, b and Table 1). Morphometry analysis showed significant changes in HW/BW ratios in Tg mice compared to NTg (Table 1) reflecting adverse cardiac remodeling followed by dilation exacerbating cardiac dysfunction in the miRNA-7 Tg mice.

**Table 1:** Age-dependent Echocardiography and Morphometric parameters for NTg and miRNA-7 Tg mice.

	3 Months (12)		6 months (12)		12 months (12)		18 months (12)	
	NTg	Tg	NTg	Tg	NTg	Tg	NTg	Tg
**Morphometry**								
HW (mg)	120 ± 2.2	140 ± 2.1*	145 ± 2.5	168 ± 4.5*	180 ± 2.6	185 ± 2.5	191 ± 2.3	179 ± 2.3*
BW (g)	25.1 ± 0.4	25.7 ± 0.52	30.5 ± 0.57	30.6 ± 0.95	34.3 ± 0.53	34.7 ± 0.59	38.3 ± 0.48	39.0 ± 0.42
HW/BW (mg/g)	4.78 ± 0.09	5.47 ± 0.15*	4.78 ± 0.16	5.52 ± 0.13*	5.25 ± 0.12	5.35 ± 0.10	5.01 ± 0.08	4.60 ± 0.07*
**Echocardiography**								
LVEDD (mm)	3.34 ± 0.06	3.44 ± 0.06	3.45 ± 0.05	3.53 ± 0.06	3.52 ± 0.06	3.90 ± 0.73*^#^	3.88 ± 0.06	4.10 ± 0.06*^#^
LVESD (mm)	1.89 ± 0.03	1.94 ± 0.05	2.07 ± 0.03	2.12 ± 0.06	2.17 ± 0.07	2.70 ± 0.07*^#^	2.63 ± 0.08	3.11 ± 0.07*^#^
PW (mm)	0.65 ± 0.04	0.65 ± 0.05	0.71 ± 0.04	0.66 ± 0.05	0.78 ± 0.06	0.50 ± 0.05*^#^	0.66 ± 0.04	0.44 ± 0.04*^#^
AW (mm)	0.83 ± 0.03	0.86 ± 0.04	0.87 ± 0.03	0.86 ± 0.05	0.94 ± 0.06	0.71 ± 0.06*^#^	0.89 ± 0.06	0.59 ± 0.06*^#^
%FS	43.4 ± 1.57	43.2 ± 1.30	40.4 ± 1.62	39.9 ± 1.37	38.3 ± 1.69	30.7 ± 1.50*^#^	32.0 ± 1.59	24.1 ± 1.54*^#^
%EF	75.7 ± 1.45	75.5 ± 1.32	71.4 ± 1.69	71.2 ± 1.52	69.2 ± 1.54	58.1 ± 1.47*^#^	61.5 ± 1.71	48.4 ± 1.78*^#^

*p < 0.001 vs. NTg, #*p* < 0.005 vs. 3 & 6 months Tg.

### miRNA-7 Tg hearts have reduced ERBB2 levels

Studies have shown that members of the epidermal growth factor receptor (EGFR) family play a key role in cardiac hypertrophy and growth response^14,30^. Since miRNA-7 targets ERBB receptor^24^, total cardiac lysates were immunoblotted for ERBB2. Significant reduction in ERBB2 expression was observed in the Tg mice compared to NTg (Fig. 2c). To provide unequivocal evidence that miRNA-7 expression targets ERBB2 in the cardiomyocytes, adult cardiomyocytes were isolated from Tg and NTg hearts and immunoblotted for ERBB2 and EGFR (ERBB1). There was significant reduction in ERBB2 expression in Tg cardiomyocytes compared to NTg (Fig. 2d), while there was no appreciable difference in EGFR (ERBB1) expression (Fig. 2d). This suggests that the adverse cardiac remodeling of dilation observed with age in miRNA-7 Tg mice could, in part be due to reduced expression of ERBB2.

### Pressure overload by transverse aortic constriction (TAC) accelerates cardiac dysfunction in miRNA-7 Tg mice

**Figure 3:**
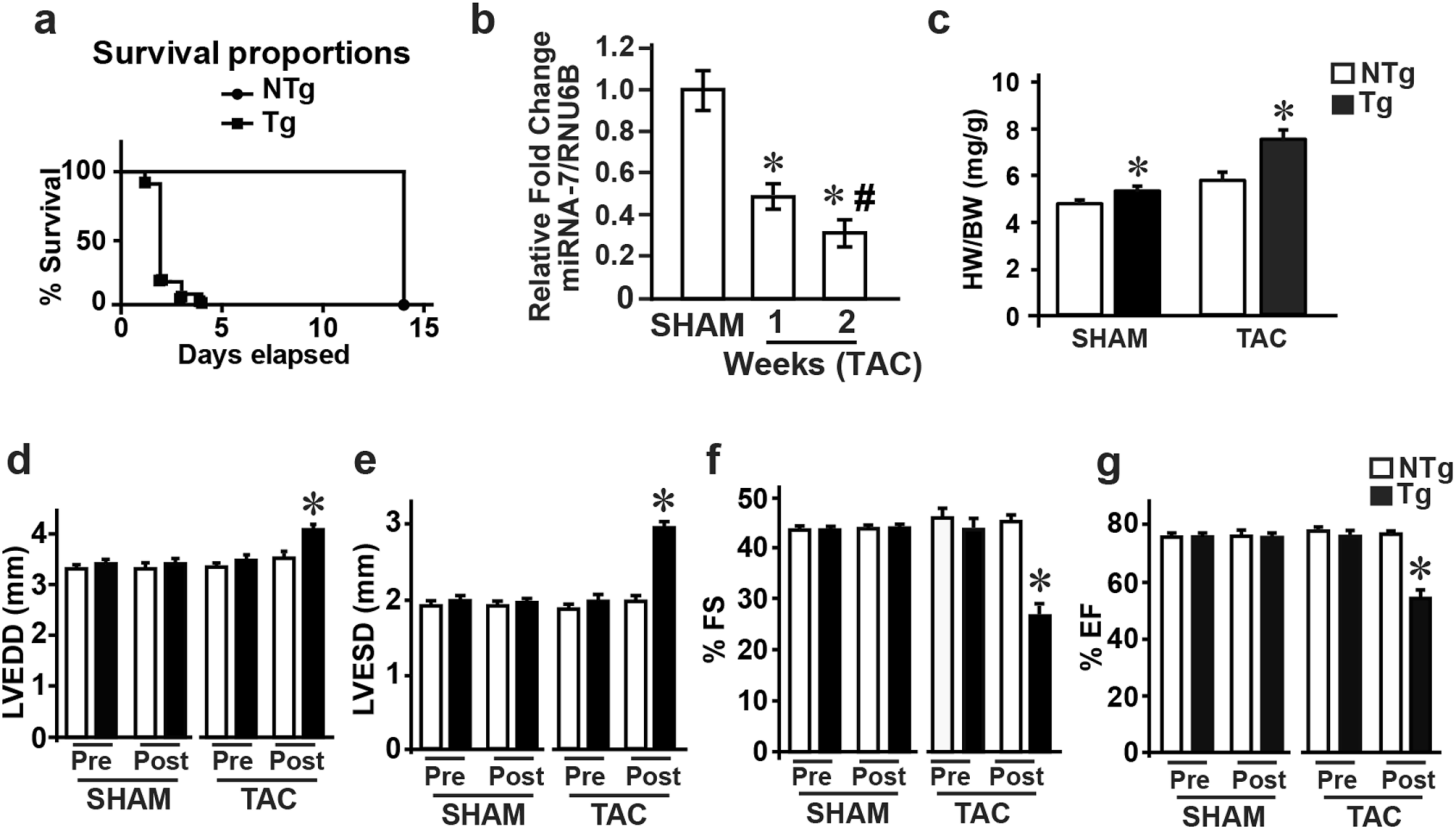
Cardiomyocyte expression of miRNA-7 exacerbates cardiac dysfunction and failure following pressure overload by transverse aortic constriction (TAC) (**a**), Kaplan–Meier survival curves for 12 months old NTg and Tg mice subjected to TAC (n = 8). (**b**), Real time PCR analysis for miRNA-7 expression from cardiac tissue of NTg mice following Sham, 1 or 2 weeks of TAC. *p < 0.001 vs. SHAM; #*p* < 0.01 vs. 1 week TAC (n = 8). (**c**), Heart weight to body weight (HW/BW) ratio of NTg and Tg following two weeks of TAC in 3 months old mice (n = 11).*p < 0.05 vs. SHAM. (**d–g**) Cardiac functional parameters of NTg and Tg mice measured by echocardiography following 2 weeks of TAC (n = 11); LVEDD (left ventricular end-diastolic dimension) (**d**), LVESD (left ventricular end-systolic dimension) (**e**), percent fractional shortening (%FS) (**f**), percent ejection fraction (%EF) (**g**). *p < 0.005 vs. Sham (NTg or Tg) and pre-TAC (NTg or Tg) (n = 11).

Since the miRNA-7 Tg mice survive normally despite cardiac dysfunction, older (12 months) miRNA-7 Tg and NTg mice were subjected to pressure overload by TAC to determine whether it would accelerate adverse remodeling. Interestingly, no Tg mice survived past 4 days following TAC, while no mortality was observed in the NTg group (Fig. 3a) showing that older miRNA-7 Tg cannot withstand cardiac stress induced by TAC. Since less is known about changes in miRNA-7 expression in response to TAC, NTg mice were subjected to TAC for 1 or 2 weeks and mRNA was isolated from these cardiac tissue and subjected to qRT-PCR. Interestingly, significant reduction in miRNA-7 levels were observed by one week of TAC which was further reduced by two weeks (Fig. 3b (normalized values) & Supplementary Fig. 1b [relative values]). To test whether younger mice would survive the cardiac stress by TAC, 3 months old Tg and NTg mice were subjected to TAC for two weeks. Gravimetric analysis showed significant increase in heart weight to body weight (HW/BW) ratio in Tg mice compared to NTg (Fig. 3c). Although there was no appreciable difference in the baseline echocardiography measurements, subjecting Tg mice to TAC resulted in acceleration of cardiac dysfunction associated with cardiac dilation compared to NTg (Table 2). Tg mice were characterized by increased left ventricular end-diastolic and -systolic dimensions (LVEDD and LVESD) post-TAC (Fig. 3e and f). Consistent with cardiac dysfunction, Tg mice displayed significant reduction in % factional shortening (%FS) and % ejection fraction (%EF) post-TAC compared to NTg (Fig. 3f and g). In contrast to the cardiac dilation observed in the Tg, NTg mice showed classical adaptive hypertrophic response to TAC as reflected by increased anterior and posterior wall thickness (Table 2). Since Tg mice undergo cardiac dilation post-TAC bypassing the adaptive hypertrophy, adult cardiomyocytes (from 3 months old mice) were isolated to evaluate in vitro cardiomyocyte contractility. Adult cardiomyocytes from NTg mice showed increased contractile response to ISO stimulation (Supplementary Fig. 1c, upper right panel (ISO) & 1d). Despite baseline cardiomyocyte contractility in Tg mice being similar to NTg (Supplementary Fig. 1c, lower left panel (baseline)), there was a significant loss in ISO-mediated cardiomyocyte contraction (Supplementary Fig. 1c, lower right panel (ISO) & 1d). This data supports the idea that miRNA-7 Tg mice are innately predisposed to cardiac dysfunction which is exacerbated in response to stress bypassing the adaptive hypertrophic response whether it is physiological (Fig. 2) or pathological (Fig. 3).

**Table 2:** Morphometric and echocardiographic parameter for NTg and miRNA-7 Tg mice with TAC (2 weeks).

Morphometry	NTg		Tg (miRNA-7)					
	Sham	TAC	Sham	TAC				
	n = 10	n = 11	n = 11	n = 11				
Heart weight (HW, mg)	125 ± 4.77	152 ± 6.98*	143 ± 5.09*	177.2 ± 10.2*^#^				
Body weight (BW, g)	26.3 ± 0.95	25.9 ± 0.80	26.6 ± 0.86	24.6 ± 0.6				
HW/BW (mg/g)	4.74 ± 0.07	5.87 ± 0.23*	5.39 ± 0.10*	7.25 ± 0.48*^#^				

Echocardiography	n = 10		n = 11		n = 11		n = 11	
	Pre	Post	Pre	Post	Pre	Post	Pre	Post
LVEDD (mm)	3.37 ± 0.03	3.38 ± 0.07	3.36 ± 0.03	3.60 ± 0.05*	3.49 ± 0.06	3.50 ± 0.07	3.49 ± 0.05	4.11 ± 0.09*^#^
LVESD (mm)	1.90 ± 0.04	1.91 ± 0.04	1.90 ± 0.05	2.02 ± 0.06*	1.97 ± 0.04	1.97 ± 0.06	1.98 ± 0.06	3.01 ± 0.08*^#^
PW (mm)	0.62 ± 0.02	0.64 ± 0.03	0.62 ± 0.01	0.80 ± 0.03*	0.63 ± 0.02	0.63 ± 0.02	0.62 ± 0.01	0.37 ± 0.03*^#^
AW (mm)	0.83 ± 0.03	0.85 ± 0.03	0.84 ± 0.05	1.11 ± 0.07*	0.88 ± 0.03	0.88 ± 0.03	0.87 ± 0.03	0.50 ± 0.03*^#^
FS (%)	43.6 ± 1.25	43.5 ± 2.12	43.6 ± 1.55	43.7 ± 1.02	43.4 ± 0.82	43.5 ± 1.25	43.6 ± 1.35	26.7 ± 1.35*^#^
EF(%)	75.9 ± 1.36	75.8 ± 2.24	75.9 ± 1.68	75.8 ± 1.17	75.6 ± 0.89	75.7 ± 1.38	75.2 ± 1.55	52.6 ± 2.12*^#^

*p < 0.001 vs. Sham NTg, #*p* < 0.005 vs. NTg Sham & TAC and Tg Sham.

### Dilation in miRNA-7 Tg mice is associated with fibrosis

**Figure 4:**
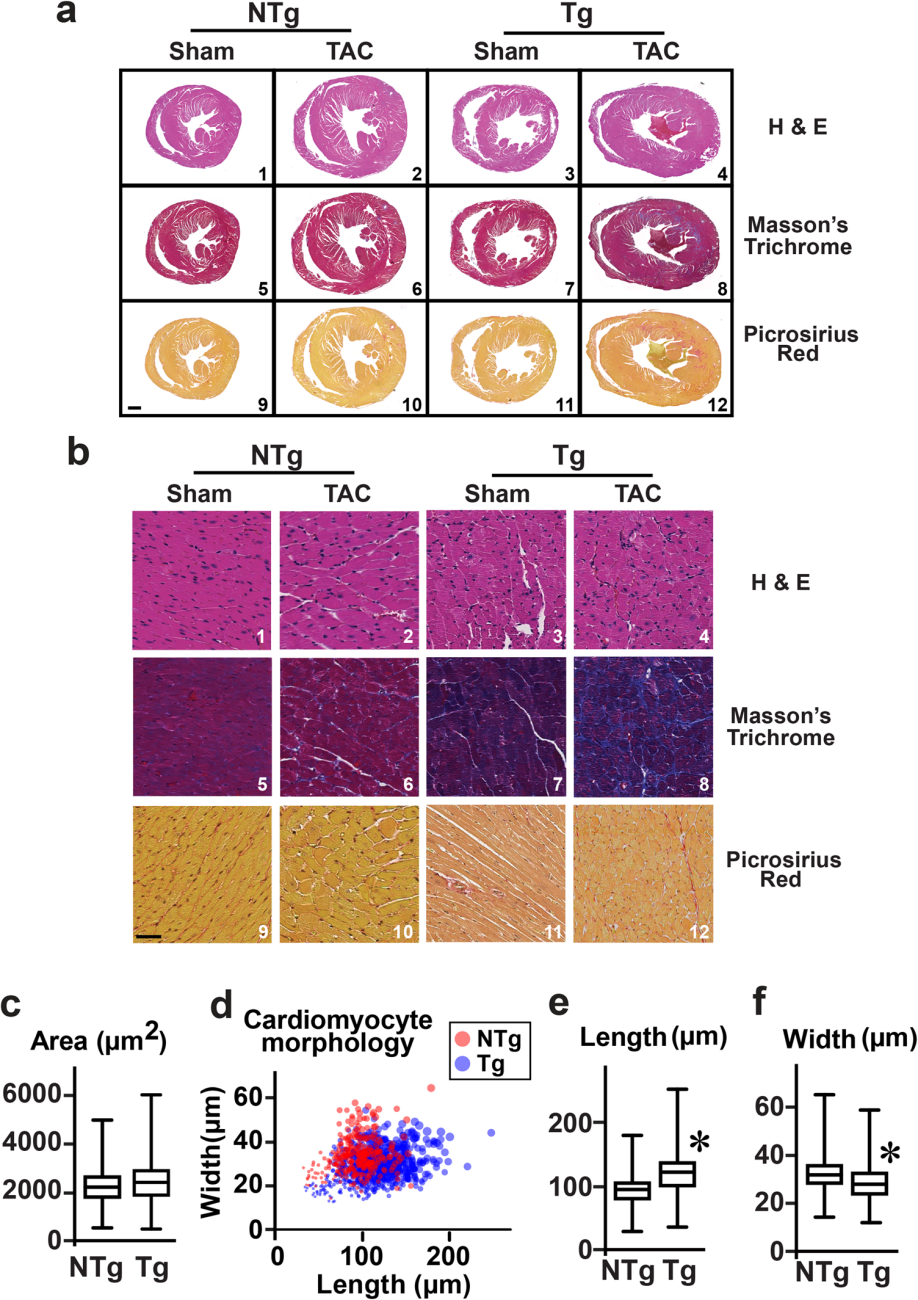
Immunohistochemistry of cardiac sections and adult cardiomyocyte morphology—Heart sections from NTg and Tg were stained with H & E, and collagen deposition was assessed using Masson’s Trichrome or Picrosirius red as indicators of fibrosis (n = 4). (**a**) Upper panel—lower magnification of the transverse sections. Scale bar 1000 µm. (**b**) Lower panel—higher magnification. Scale bar 200 µm (n = 4). Adult cardiomyocytes isolated from NTg and Tg were used for measuring cellular morphology using Image Pro 10 (**c**) cardiomyocyte area (µm^2^), (**d**) cardiomyocytes morphology across measures of length and width (µm), (**e**) length and (**f**) width. *p < 0.001 vs. NTg (> 400 cells).

Because miRNA-7 Tg mice undergoes cardiac dilation instead of the adaptive hypertrophy in response to TAC, immunohistochemistry (H & E, Masson’s trichrome and picrosirus red staining) was performed to determine whether accelerated cardiac dilation is associated with fibrosis. Consistent with increased HW/BW ratio, miRNA-7 Tg sham was characterized by a slightly larger heart than NTg sham (Fig. 4a, panels 1 & 3). NTg heart displayed classical adaptive cardiac hypertrophy following TAC (Fig. 4a, panel 2), while Tg showed marked increase in size associated with larger lumen (Fig. 4a, panel 4) upon TAC reflecting cardiac dilation consistent with echocardiography analysis (Fig. 3). Higher magnification H & E staining showed marked disorganization of the cardiomyocytes even in sham miRNA-7 Tg (Fig. 4b, panel 3) compared to NTg (Fig. 4b, panels 1 & 2). miRNA-7 Tg showed elevated Masson’s Trichrome and picrosirius red staining in both sham and TAC hearts (Fig. 4b, panels 7, 8, 11 and 12) compared to NTg (Fig. 4b, panels 5,6,9 and 10) reflecting increased fibrosis. These findings show that elevated deposition of interstitial collagen and increased cardiac fibrosis^31^ may underlie the exacerbated cardiac dysfunction observed in miRNA-7 Tg mice. Since miRNA-7 Tg are characterized by slightly larger hearts, cardiomyocytes were isolated from 3 months old NTg and Tg mice to assess whether miRNA-7 Tg heart have eccentric hypertrophy. Analysis of the isolated cardiomyocytes showed only a trend towards increased cardiomyocyte area in miRNA-7 Tg compared to NTg but was not significant (Fig. 4c). Given this observation, comprehensive analysis on cardiomyocyte morphology (see methods) was assessed relative to their respective length and width. miRNA-7 Tg cardiomyocytes showed a propensity towards increased length compared to NTg (Fig. 4d). Consistently, measurements of individual cardiomyocytes showed that miRNA-7 Tg mice have increased length (Fig. 4e) and decreased width (Fig. 4f) compared to NTg supporting the idea of baseline eccentric hypertrophy in miRNA-7 Tg mice that pre-disposes them towards increased fibrosis and dramatic dysfunction following TAC.

### Proteomic and networking analysis in miRNA-7 Tg hearts shows alterations in mitochondrial components

**Figure 5:**
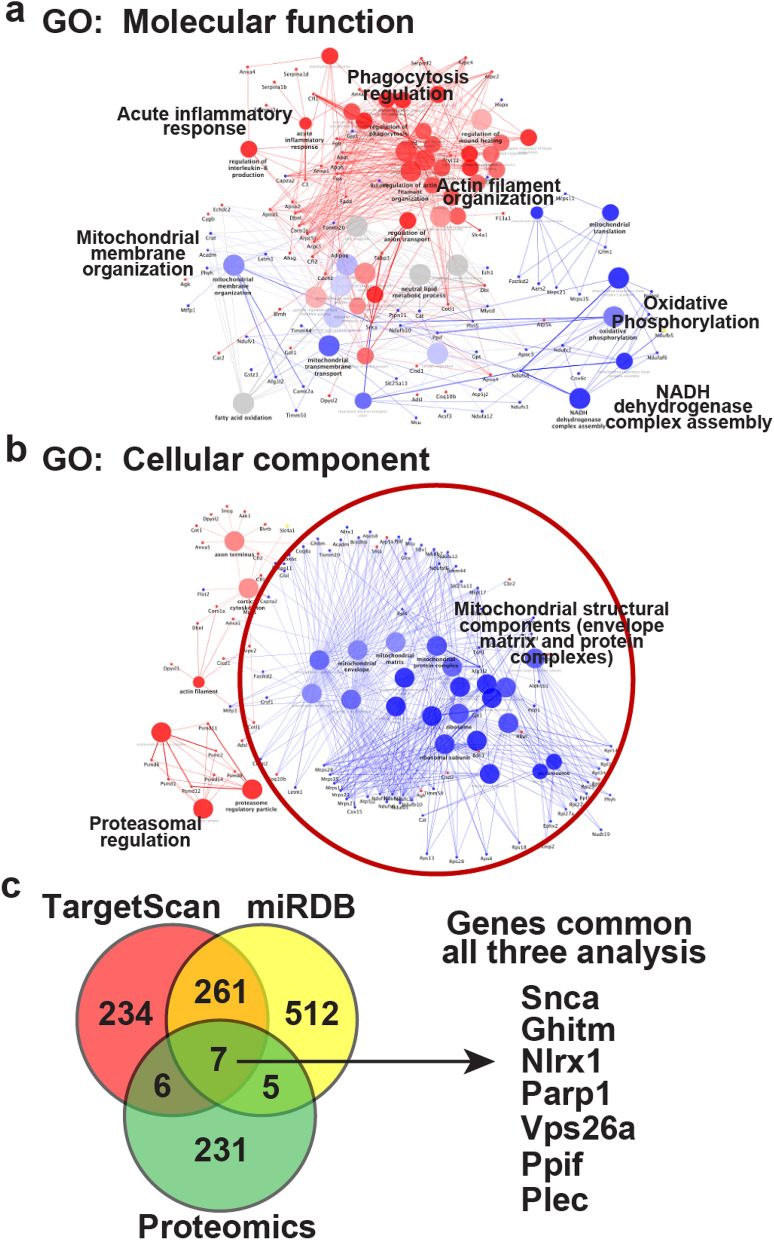
Cardiac proteomics and network analysis of miRNA-7 targets in NTg and Tg hearts (**a**), Gene ontology (GO) functional analysis using the significantly altered proteins in the Tg versus NTg from cardiac proteomics shows unique molecular functional changes in the Tg wherein phagocytosis, actin remodeling and acute inflammation are upregulated (red color coding). While, mitochondrial structure and function are downregulated (blue color coding) (https://apps.cytoscape.org/apps/cluego). (**b**), GO cellular component analysis shows upregulation of proteosomal components (red color) while, majority of the pathways are associated with downregulation of mitochondrial structural components. (**c**), Venn diagram showing an overlap of predicted targets (from prediction databases TargetScan and miRDB) and truly altered proteins identified in the cardiac proteomic studies from NTg and Tg hearts. Seven common proteins identified are shown in right that are all downregulated in the miRNA-7 Tg mice compared to NTg.

Although we observed loss of ERBB2 expression in the cardiomyocyte following miRNA-7 expression, it is well known that miRNAs can target multiple transcripts, wherein the overall phenotype is a composite effects of all changes. In recognition, we performed proteomics on 3 month old hearts from miRNA-7 Tg and NTg mice (n = 3) with the rationale that molecular changes precede the phenotypic effects given that cardiac function is still preserved in miRNA-7 Tg mice and similar to NTg (Fig. 2a). Cardiac lysates were resolved on SDS-PAGE, in-gel trypsinized and subjected to LC–MS mass spectrometry analysis. Analysis showed that > 249 proteins were significantly altered with majority of them being downregulated in miRNA-7 Tg hearts compared to NTg including ERBB2 despite preserved cardiac function. These set of altered proteins in miRNA-7 Tg hearts were used for gene ontology (GO) networking analysis (see methods) to assess for their role in molecular function and cellular component enrichment. Molecular functional analysis of the altered proteins in miRNA-7 Tg mice showed upregulation of pathway involved in phagocytosis and actin remodeling consistent with observation of eccentric hypertrophy. While, there was downregulation of oxidative phosphorylation function associated with loss in NADH dehydrogenase complex (Fig. 5a and Supplementary Fig. 2). Further examination of GO cellular components showed marked upregulation of proteasomal pathways and significant downregulation of mitochondrial matrix and envelope components in miRNA-7 Tg hearts compared to NTg (Fig. 5b and Supplementary Fig. 3).

Since global proteomics identified > 249 proteins to be altered in the miRNA-7 Tg hearts, two miRNA target predicting databases (miRDB and TargetScan) were used to compare the predicted targets versus the actual representations of proteins altered in the Tg hearts. The predicted and the proteomics data in the Venn diagram shows that among > 249 altered proteins, only 18 proteins represent the predicted targets of miRNA-7 that are significantly altered in miRNA-7 Tg hearts compared to NTg. Interestingly, among these 18 proteins only 7 (Supplementary Table 1) were common to both the miRDB and TargetScan prediction databases and were significantly downregulated in the cardiac proteomics study (Fig. 5c]. Among the 7 downregulated proteins, two of targets GHITM (Growth hormone-inducible transmembrane protein) and PPIF (Cyclophilin F) may play a critical role in the mitochondrial integrity and cristae organization^32^. This would be consistent with the GO cellular component analysis showing significant downregulation of mitochondrial matrix and envelope proteins that may underlie mitochondrial dysfunction.

### Validation of proteins identified by unbiased proteomics

**Figure 6:**
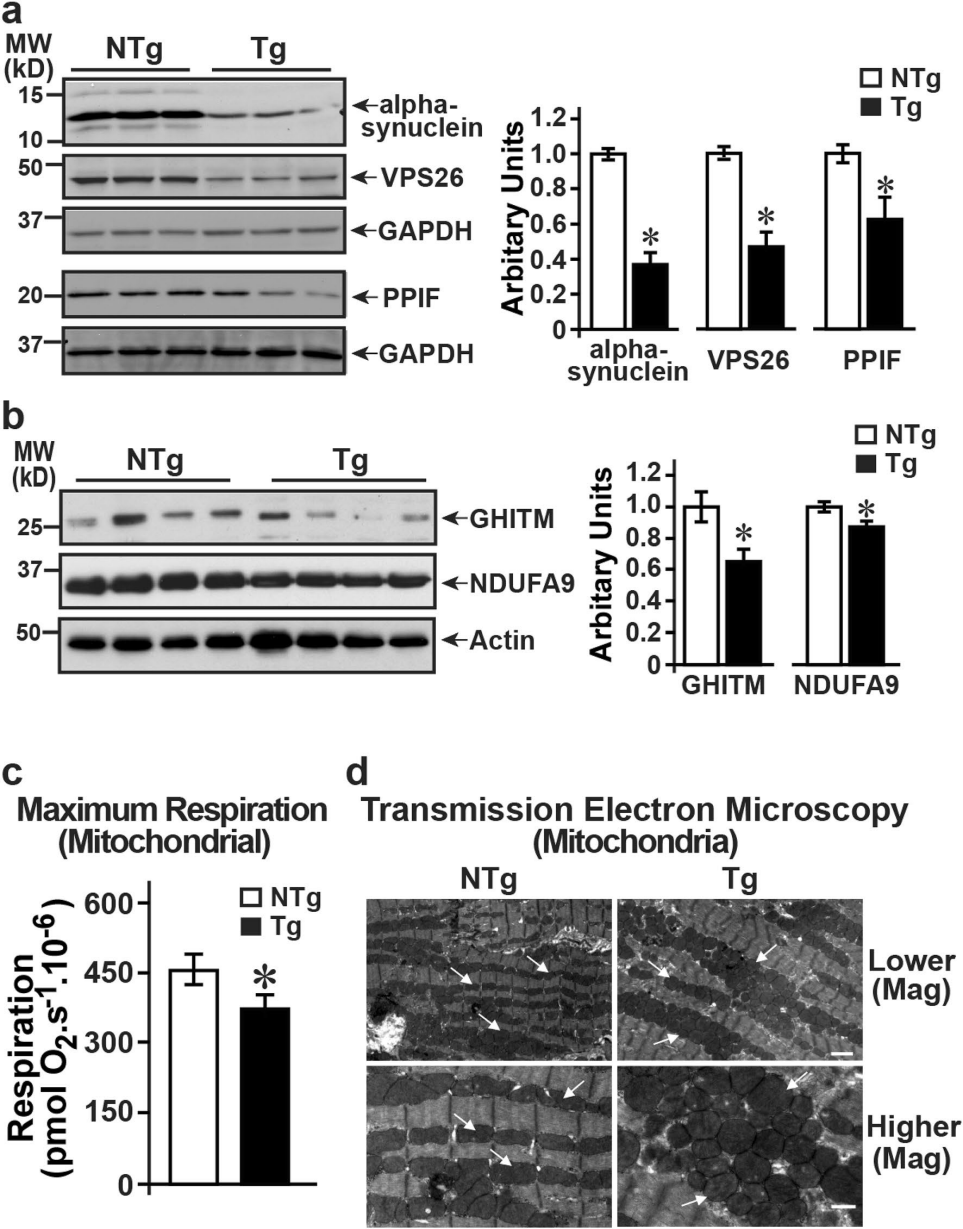
Validation of proteomic studies, and mitochondrial structure–function ananlysis (**a**), Cardiac lysates (100 µg) from an independent set of NTg and miRNA-7 Tg mice were immunoblotted with anti-alpha synuclein, anti-VPS26, or anti-PPIF and stripped and reblotted with GAPDH (left panels). Right panel, cumulative data *p < 0.001 vs. NTg (n = 7). (**b**) Immunoblotting was performed on cardiac lysates (100 µg) using anti-GHITM and the blots were stripped and reblotted with anti-NDUFA9 followed by actin (left panels). Right panel, cumulative data *p < 0.001 vs. NTg (GHITM) & *p < 0.01 vs. NTg (NDUFA9) (n = 4). (**c**) Mitochondria isolated from miRNA-7 Tg and NTg underwent high resolution oxygen consumption assessment using the Oroboros high resolution respirometry wherein maximum respiration is presented as a measure of mitochondrial function. *p < 0.01 (n = 3). (**d**) Transmission electron microscopy of the heart sections from NTg and Tg shows marked alteration in mitochondrial ultra-structure. NTg mice are characterized by well-organized mitochondria aligned with sarcomeres. Tg mitochondria are disorganized and rounded in shape reflecting alterations in mitochondrial structural components (n = 5). Upper panel—lower magnification (scale bar—2 μm); Lower panel—higher magnification (scale bar—1 μm).

Our global proteomics identified a set of proteins that were consistently identified as targets of miRNA-7. Validation studies were performed by western immunoblotting on cardiac lysates prepared from the hearts of an independent set of NTg and miRNA-7 Tg mice. Immunoblotting studies showed that α-synuclein, VPS26, and PPIF (cyclophilin F) were all significantly downregulated in the miRNA-7 Tg mice compared to NTg (Fig. 6 a, left and right panel). Furthermore, immunoblotting studies showed that GHITM, a molecule critical for cristae structures is also significantly downregulated in miRNA-7 Tg mice compared to NTg (Fig. 6b, left and right panel). These observations are consistent with the proteomics studies and a reflection of them being targets of miRNA-7. Since proteomics and networking analysis showed that there is significant reduction in the NADH dehydrogenase complex, western immunoblotting was performed on a key component of NADH dehydrogenase NDUFA9. Immunoblotting showed mild yet significant reduction of NDUFA9 in the Tg hearts compared to the NTg (Fig. 6b, left and right panel) suggesting a potential for mitochondrial dysfunction. To directly determine whether these changes in proteins underlie mitochondrial dysfunction, mitochondria were purified from isolated adult cardiomyocytes from NTg and miRNA-7 Tg. The purified mitochondria were evaluated for oxygen consumption using Oroboros oxygraph high-resolution respirometry. Comprehensive functional analysis showed that there was significant reduction in the maximum respiration of the mitochondria from the miRNA-7 Tg mice compared to NTg (Fig. 6c & Supplementary Fig. 4 [representative oxygen consumption curves from NTg and miRNA-7 Tg]). These observations show that expression of miRNA-7 in the cardiomyocytes leads to unique targeting of proteins that in turn leads to mitochondrial dysfunction which may underlie the observed adverse remodeling in the miRNA-7 Tg mice.

### Altered mitochondrial ultrastructure in miRNA-7 Tg hearts

Given that proteomic studies, western immunoblotting and functional analysis showed mitochondrial dysfunction, cardiac mitochondrial structure was evaluated in the miRNA-7 Tg mice. Since these studies were performed on 3 months old mice, transmission electron microscopy was performed on the 3 months old miRNA-7 Tg and NTg hearts. Significant difference in structure and morphology of the mitochondria was observed in the miRNA-7 Tg hearts compared to NTg (Fig. 6d). Mitochondria are disorganized in the miRNA-7 Tg hearts and are round with ultrastructure showing lack of cristae that seem to be undergoing fission. This observation suggests that changes in mitochondrial structure as well as its dysfunction, in part may lead to adverse cardiac remodeling.

## Discussion

Heart meets the increasing demands of the body by an adaptive hypertrophy in response to aging (considered physiological) and stress like pressure overload (that is pathological)^33^. The current study shows that cardiomyocyte expression of miRNA-7 leads to cardiac dilation that bypasses the classical adaptive hypertrophy to increasing mechanical overload. This is supported by the findings that miRNA-7 Tg mice undergo age-based cardiac dilation in contrast to physiological adaptive hypertrophy observed in control NTg. The younger miRNA-7 Tg mice despite having normal cardiac function, directly transition to dilation following TAC indicating a unique role for miRNA-7. In this context, adult cardiomyocytes from miRNA-7 Tg mice are morphologically longer than the NTg with a potential for eccentric hypertrophy which now predisposes the hearts towards dilation with aging or stress. Cardiac dilation in miRNA-7 Tg mice post-TAC is associated with increased fibrosis as assessed by Masson trichrome staining and collagen deposition by picrosirius red. miRNA-7 expression leads to significant loss of ERBB2 in the cardiomyocytes instead of the EGFR that is observed in cancer cells^24^. Proteomic analysis on the miRNA-7 Tg hearts showed that > 249 proteins are significantly altered with majority of the proteins being downregulated including those that play a role in mitochondrial integrity. Consistent with this observation, high resolution electron microscopy showed rounded and disorganized mitochondria associated with reduced maximum respiration that in part, could underlie the exacerbated cardiac dysfunction observed in miRNA-7 Tg mice.

Cardiomyocyte-specific miRNA-7 expression results in abrogation of ERBB2 expression in cardiomyocytes underlying cardiac dilation and a phenotype that is consistent with the conditional ERBB2 knockout mice^13,34^. Despite significant ERBB2 abrogation in cardiomyocytes, total cardiac lysates show minor levels of ERBB2 expression reflecting contributions from other cell types in the heart. Studies in cancer cells have shown that miRNA-7 reduces EGFR (ERBB1) expression^24^ but miRNA-7 expression in the cardiomyocytes does not alter EGFR but abrogates ERBB2 expression. This potentially reflects on cell-specific regulation of ERBB1 and ERBB2 despite containing the miRNA-7 targeting leader/seed sequence. Such a unique regulation could be due to the repertoire of cell-specific mRNA binding proteins that would mask the miRNA binding site on mRNA^35,36^. This critical component has been overlooked in miRNA studies as expression of different RNA binding proteins in a given cell could contextually change the effects of miRNA on target protein expression^36^. This supports the idea that cell-specific effects could be achieved by miRNA targeting overcoming non-specific secondary effects which though exciting, needs more comprehensive global studies.

miRNA-7 Tg mice display normal cardiac function at younger age but transitions to dilation with age instead of the adaptive cardiac hypertrophy. Our studies show that miRNA-7 Tg mice are characterized by elongated cardiomyocytes that may underlie eccentric hypertrophy as supported by mild yet significant increase in cardiac mass. This unique phenotype of baseline eccentric hypertrophy may not only cause the right ventricular remodeling due to volume overload^37–39^ with age but may predispose the hearts towards adverse remodeling in response to aging or stress. Although age-based hypertrophic response is considered physiological^40^ but however, it is not reversible^1,2^. This observation suggests that this response in part, could be mediated by ERBB2 as miRNA-7 Tg mice have reduced ERBB2 expression. In this context, chemotherapeutics including anti-ERBB2 agents leads to cardiac dilation^28,41^ of the otherwise healthy hearts suggesting a role for ERBB2 pathways in maintaining dynamic cardiac hypertrophic responses. This is consistent with the cardiac dilation and accelerated cardiac dysfunction observed in the Tg mice following expression of miRNA-7 that reduces ERBB2 expression underlying the observation of bypassing the adaptive hypertrophic response post-TAC. Such a role is supported by the observation of cardiac dilation in young conditional ERBB2 knockout mice^30^ and significant cardiac hypertrophy with no failure in mice with cardiomyocyte-specific overexpression of ERBB2^14^. Although role of ERBB2 in embryonic development^9^, cardiomyocyte de-differentiation and proliferation^42^ is recognized, our current study shows that it does not alter proliferation in adult hearts (Supplementary Fig. 5) and elucidates a role for ERBB2 in adaptive hypertrophic response that maintains dynamic homeostasis.

Global proteomics was performed in recognition that miRNA-7 could target other transcripts in addition to ERBB2 which together would determine net adverse remodeling outcomes. Although > 249 proteins were significantly altered, only 18 of the predicated targets (using miRDB and TargetScan) were present in our cardiac proteomics (Supplementary Table 1). Among these 18 proteins only 7 were common between miRDB and TargetScan prediction database and all of them were downregulated (Supplementary Table 1). However in addition to these target proteins, functional networking analysis revealed downregulation of NADH dehydrogenase complex proteins and immunoblotting for the key protein NDUFA9 of the NADH dehydrogenase complex showed mild yet significant downregulation. Such an observation is supportive of the ever increasing appreciation that a large repertoire signaling network proteins are altered by miRNAs^43^. Consistent with the downregulation of NADP dehydrogenase complex, there was significant impairment in mitochondrial function as measured by maximum respiration in the miRNA-7 Tg mitochondria compared to NTg. These studies show that miRNA-7 expression not only alters mitochondrial structure but also mediates dysfunction that underlies the adverse cardiac remodeling.

Analysis of these 7 target proteins show that except for poly(ADP-ribose)polymerase-1 (PARP1) whose role is known in cardiovascular disease^44^, nothing is known about the roles of other proteins in cardiac function and regulation. PARP1 inhibition is considered beneficial in ischemia–reperfusion and given its downregulation in the Tg suggests it may not play a determinant in the observed phenotype. Given that very little is known about these altered proteins, validation studies were performed by immunoblotting that showed that SNCA (alpha-synuclein), VPS26, PPIF and GHITM were decreased in the miRNA-Tg hearts compared to NTg. SNCA accumulation is thought to underlie sympathetic denervation in patients with Parkinson Disease^45^ but its downregulation in miRNA-7 Tg indicate that it may not contribute towards adverse cardiac remodeling. Networking analysis of the altered proteins in the Tg mice showed marked increase in actin cytoskeleton and intracellular trafficking proteins which could be a reflection of compensatory effects of VPS26a downregulation that plays a key role in endosomal cargo sorting^46,47^. Plectin gene (Plec1) polymorphism is known to be associated with hypertrophic cardiomyopathy in humans^48^ and underlie muscular dystrophy due to altered interactions of mutant PLEC1 protein with cytoskeleton^49^. Despite the loss of PLEC1 protein in miRNA-7 Tg mice, there was no appreciable disorganization of the sarcomeres and the mice underwent cardiac dilation instead of hypertrophic myopathy observed with Plec1 mutations^48^.

Three of the downregulated proteins in miRNA-7 Tg hearts are localized to mitochondria (GHITM, NLRX1 and PPIF). PPIF codes for cyclophin F whose inhibition is considered to be beneficial^50^ but is already downregulated in miRNA-7 Tg mice indicating that it may not be a player in the adverse remodeling. However, less is known about the roles of NLRX1 or GHITM. NLRX1 is mitochondrial targeted NOD-like receptor family member that plays a role in anti-viral immunity^51^ by binding to mitochondrial antiviral signaling protein (MAVS)^52^. Consistent with its loss, our molecular functional analysis shows upregulation of acute inflammatory response indicating a potential role for NLRX1 in the miRNA-7 Tg hearts. Perhaps the most intriguing protein in our proteomics study is the GHITM (Growth hormone-inducible transmembrane protein) also known as transmembrane BAX inhibitor motif containing protein 5 (TMBIM5)^32^. GHITM is a member of the BAX inhibitor containing (TMBIM) family that localizes to the inner mitochondrial membrane (IMM) where it plays a role in apoptosis by mediating alterations in mitochondrial morphology and cytochrome c release^32^. Although the role for GHITM is not known in cardiac systems, GHITM is considered to maintain the cristae organization and its downregulation results in mitochondrial fragmentation potentially through fusion of the cristae structures. Consistent with proteomics studies, immunoblotting showed significant downregulation of GHITM that may underlie structural disorganization of mitochondria in the miRNA-7 Tg heart. The observation of rounded and disorganized mitochondria in Tg mice suggests a key role for GHITM in maintaining mitochondrial integrity. Furthermore, it is also known that loss in GHITM expression leads to release of pro-apoptotic protein cytochrome C^53^ and could potentially underlie the fibrosis observed in the Tg mice hearts even at baseline which is exacerbated following TAC (Fig. 4b). Also, GHITM is thought to be responsible for cross-linking cytochrome C to IMM thereby, delaying the release of cytochrome C. The proteomics study suggests that GHITM may play a role in adverse cardiac remodeling in the Tg mice by modulating fibrosis through sequestration of cytochrome C and regulating mitochondrial integrity bringing-to-fore, a yet to be understood role of GHITM in cardiac remodeling.

Our comprehensive study shows the miRNA-7 expression leads to adverse cardiac dilation instead of adaptive hypertrophy that, in part could be mediated by loss in ERBB2 and GHITM expression. The observation of ERBB2 targeting by miRNA-7 instead of EGFR1 observed in cancer cells suggests unique cell specific mechanisms of regulation. Such an idea is further supported by our proteomics study wherein beta-arrestin 1 despite being a bonafide target^54^ of miRNA-7 is not altered in the cardiomyocytes indicating that cellular studies to assess miRNA targeting may not reflect the unique cardiomyocyte specific regulation. Together our study unravels an interesting aspect that despite multiple predicted targets for miRNA, only a few targets are significantly altered which may contribute to the overall phenotype. However, this also suggests that the idea to use miRNA-7 as a potential anti-cancer therapeutic^26^ may have to be carefully determined given the adverse remodeling observed with cardiomyocyte expression of miRNA-7, an observation reminiscent of anthracycline mediated cardiotoxicity.

## Methods

### Experimental animals

All mice used in the studies are in C57Bl/6 background. Transgenic (Tg) mice with cardiomyocyte specific expression of miRNA-7 and littermate controls were used in the study. Since there is growing support for the use of miRNA-7 to treat cancers, our studies were initiated to evaluate the role of miRNA-7 in the hearts in pathology and physiology.

### Generation of miRNA-7 Tg mice

Mice with cardiomyocyte-specific expression of mature human miR-7–1-5p sequence (5’- UGGAAGACUAGUGAUUUUGUUGU-3’) under the control of alpha-MHC promoter were generated at the Transgenic targeting facility (Case Western Reserve University) in the C57BL/6 background. Four positively identified founders were bred with C57BL/6 mice. After breeding for three generations, the F3 pups were assessed for expression of miR-7–1-5p in the hearts. Only one founder line showed expression of miR-7 which was used in our studies. The Kaplan–Meier procedure was used to calculate survival with time and the data is as % survival over days. Arrive statement: We would like to state that the study has been designed and performed as per the ARRIVE guidelines including the randomization, blinding of the operator, outcomes and statistical methods. Importantly, given that cardiac disease and deleterious remodeling occurs in both male and females, we have included both genders of mouse in our study. Animals were handled according to the approved protocols and animal welfare regulation of IACUC at Cleveland Clinic following the approved NIH guidelines.

### Transverse aortic constriction (TAC) and ERBB2 inhibitor studies

miRNA-7 Tg mice and littermate controls were subjected to pressure overload by performing transverse aortic constriction (TAC) surgery. Briefly, following anesthesia with ketamine and xyalzine, the mouse was connected to a rodent ventilator and surgery performed as previously described^11^. Sham operated animals underwent the similar procedure except for the aortic constriction.

### RNA isolation and northern blotting

RNA isolation and northern blotting was performed as described previously^55^. Briefly, left ventricular tissue was homogenized using TRIZOL (Invitrogen) and integrity of the RNA assessed by spectroscopic analysis. 20 μg of RNA was size-fractionated by denaturing formaldehyde gel electrophoresis, transferred to nylon membrane and cross-linked. The membrane was stained with 0.5% methylene blue to visualize the transferred RNA and for equal loading. The intensely stained 28 s and 18 s rRNA bands were used as the internal molecular weight markers for the RNA. The membranes were de-stained with diethylpyrocarobonate (DEPC)-treated water and hybridized with (^32^P) labeled mature miRNA-7 probe. Following hybridization, the filters were washed under stringent conditions to visualize miRNA-7 transcripts by autoradiography.

### Quantitative real time-PCR

Two μg of total RNA was used for reverse transcription (RT) with RT kit (Applied Biosystems) using specific primer sets supplied by Taqman kit for miRNA-7–1-5p. Quantitative real-time PCR (qPCR) was performed using cDNA with Taqman real time mix in a BioRad iCycler machine. The fold change in the miRNA levels was determined using RNU6B as an internal control. ΔΔCt method was used to compare the miRNA-7 Tg mice with littermate controls. The 2-ΔΔCT method was used as relative quantification strategy for qPCR data analysis^56^.

### Echocardiography

Echocardiography was performed on anesthetized mice using a VEVO 770 and VEVO 2100 (VISUALSONICS) echocardiographic machine as previously described^57^. M-mode recording was used to obtain cardiac functional parameters.

### Cardiac lysate and Western Immunoblotting

Cardiac lysates were isolated as previously described^58^. Briefly, the cardiac tissue was homogenized in lysis buffer (1% Nonidet P-40 (NP 40), 20 mM Tris–Cl pH 7.4, 300 mM NaCl, 1 mM EDTA, 20% glycerol, 0.1 mM PMSF, 10 μg ml^−1^ each of Leupeptin, and Aprotinin). The cardiac lysates were centrifuged 38,000 × g for 25 min at 4ºC and the supernatant used for western analysis. 100 µg of total cardiac lysates were resolved on a SDS-PAGE gel, transferred to PVDF membranes (BIO-RAD) and western immunoblotting was performed using anti-ERBB2 (Neu) antibody (1:500) (Santa Cruz), anti-alpha-synuclein (1:300) (Abcam), anti-VPS26 (1:500) (Invitrogen), anti-PPIF (1:200) (ThermoFisher), anti-GHITM (1:200) (ThermoFisher), anti-NUDFA9 (1:10,000), anti-GAPDH (1:5000) (Abcam), anti-actin (1:5000) (Sigma) followed by chemi-luminescence. Similarly, 50 µg of adult cardiomyocyte lysates were used for ERRB2 western immunoblotting studies. Densitometry analysis was carried out using the NIH image J software.

### Isolation of adult cardiomyocytes, contractility and morphometry

Adult cardiomyocytes were isolated as previously described^57^. Briefly, excised hearts from anesthetized mice were immediately cannulated with 20-gauge needle and mounted to perfusion apparatus. The perfusion buffer contained 113 mM NaCl, 4.7 mM KCl, 0.6 mM KH2PO4, 0.6 mM Na2PO4, 1.2 mM MgSO4, 0.5 mM MgCl2, 10 mM HEPES, 20 mM D-glucose, 30 mM taurine and 20 μM Ca2 + at pH 7.4. Following perfusion for 4 min, 150 units/ml of type II collagenase was perfused for 15 min. All the solutions were maintained at 37°C and continuously bubbled with 95% O2 and 5% CO2. Left ventricular tissue was separated from the atria and right ventricle, minced, and digested in perfusate for 15 min. The digested heart was filtered through 200 μm nylon mesh, placed in a conical tube, and spun at 100 rpm to allow viable myocytes to settle. Serial washes were used to remove nonviable myocytes and digestive enzymes, and the adult myocytes were collected. Isolated cardiomyocytes were used for in vitro isoproterenol stimulated cardiomyocyte contractility using the IonOptix System (Myopace, Milton, MA) as previously described^57^ and subset were lysed in the lysis buffer for western immunoblotting. Isolated adult cardiomyocytes were plated on Laminin (100 µg/ml) coated plates and fixed after 3 h. The images were acquired using a Leica DMI6000SD inverted microscope (Leica Microsystems, GmbH, Wetzlar, Germany) equipped with a Leica DFC7000 T camera. The images were processed by detecting the cardiomyocytes using a pixel classifier in QuPath v0.2.3^59^. The identified cardiomyocytes were classified into three groups based on their shape and size to identify healthy cardiomyocytes, clumps and dying cells. Mask image of healthy cardiomyocytes was exported from QuPath and analyzed using Image-Pro 10 (Media Cybernetics), wherein measurements were performed for individual cells for area, ferret length and ferret width.

### Immunohistochemistry

Immunohistochemistry was performed as previously described^60^. Excised hearts were fixed in 5% paraformaldehyde for 24 h, processed for embedding and sectioning at the institutional imaging core. The sections were stained using standard protocol of the imaging core for H & E, Masson’s trichome and Picrosirius Red. Measurement of dilated heart area and area of heart tissue in the transverse sections of the heart were performed using the Image Pro-Plus image and analysis software (Media Cybernetics, Rockville, MD) available within the Cleveland Clinic institutional imaging core. Dilation and heart area were measured in the miRNA-7 Tg and their littermate controls allowing for the Image Pro-Plus software to assess the area of dilation.

### Proteomics, network analysis and miRNA predicted target analysis

Cardiac lysates from 3 months old miRNA-7 Tg and littermate controls (n = 3) were resolved on 10% SDS-PAGE gel and stained with coommassie blue. The stained gels were given to the proteomics core facility within Lerner Research Institute, Cleveland Clinic. In gel digestion was performed and the digested proteins from these sample sets were injected for LC–MS. Relative abundance of the proteins were assessed based on the intensities of the observed peptides. All proteins that have differences in expression of more than two fold were used for the networking analysis. The list of statistically significant unique set of proteins differentially expressed between miRNA-7 Tg versus littermate control were used to query Gene Ontology (GO)—Biological function, Molecular Function and Cellular component database using the ClueGo Cytoscape^61^ (https://apps.cytoscape.org/apps/cluego). The following ClueGo parameters were set wherein, Go Term Fusion would select only display pathways with p values ≤ 0.05; GO tree interval at all levels were set at a term minimum of 3 genes with a threshold of 4% of genes/pathway and a kappa score of 0.42. Gene ontology terms are presented as nodes and clustered together based on the similarity of genes present in each term or pathway. Node size is proportional to the p value for the GO term enrichment. Proteins are presented as circles wherein, red circles indicate upregulated proteins and blue circles indicate downregulated proteins associated with one or more processes. Predicted targets of miRNA-7 were identified using the publicly available miRNA prediction tool miRDB^62^ and TargetScan^63^. Analysis of predicted targets for miRNA-7 using miRDB and TargetScan tools showed 758 and 508 targets respectively. These predicted target proteins were compared with our proteomics data showing differential expression of proteins in miRNA-7 Tg versus littermate controls. A Venn diagram was constructed to show targets that are common between predicted versus experimentally identified target proteins. These set of common proteins present in the proteomics data and prediction tools are represented in the Supplementary Table 1.

### Mitochondria isolation and oxygen consumption studies using Oroboros high resolution respirometry

Mitochondria was isolated from adult cardiomyocyte of miRNA-7 Tg and NTg mice using the following published protocol^64,65^ with modifications. Briefly, the isolated adult cardiomyocytes were dounced in lysis buffer (250 mM sucrose, 20 mM HEPES-NaOH (pH 7.9), 10 mM KCl, 1.5 mM MgCl2, 1 mM EDTA and protease and phosphatase inhibitor cocktails (Sigma)). Following douncing, the lysates were centrifuged at 800 X *g* at 4 °C for 10 min to remove nuclei and cellular debris. The collected supernatant was centrifuged at 10,000 X g at 4 °C for 20 min. The pellet was washed once with lysis buffer and following the wash, the isolated mitochondrial pellet was suspended in mitochondrial respiration medium, MiR05. A fraction of the mitochondria was used for measuring protein content so that equal mitochondrial content was added to each of the chamber of the respirofluorometer to measure responses to substrates, inhibitors and uncoupler. Oxidation coupled to phosphorylation was quantified in response to substrates for complexes I and II and ADP. Mitochondrial substrates, malate and pyruvate, were added to generate NADH, a complex I substrate. Malate was added as a source of oxaloacetate to maintain continued oxidation of pyruvate to acetyl CoA. Succinate was then added as a complex II substrate. Maximal oxidative capacity, or maximum respiration, was measured by the addition of the protonophore trifluoromethoxy carbonylcyanide phenylhydrazone (FCCP) followed by rotenone to inhibit electron flow across complex I and antimycin A to determine non-mitochondrial residual oxygen consumption rate. Uncoupled complex IV oxidation rate was calculated using sodium azide followed by tetramethyl phenylene diamine (TMPD) and ascorbate. Oxygen concentration and flow rates were recorded at 2 s intervals to measure oxygen consumption rates using DatLab2, Oroboros (Innsbruck, Austria). Oxygen consumption was corrected for residual oxygen consumption. Data were generated from at least 3 biological replicates after calibration of the oxygen sensors and instrument background corrections. All data were expressed as oxygen consumption in pmol.sec-1 normalized to protein content to allow for comparisons across experiments.

### Transmission electron microscopy

Transmission Electron microscopy on heart tissue was performed at the Institutional Imaging Core. Heart sample was fixed in 2.5% glutaraldehyde/4% paraformaldehyde in 0.2 M sodium cacodylate buffer overnight at 4º C. Sample was washed three times, 5 min each with 0.2 M sodium cacodylate buffer (pH 7.3). Following the wash, cold water dissolved 1% Osmium Tetroxide was added, followed by sodium cacodylate buffer wash and rinsed with Maleate buffer (pH 5.1). The tissue was stained with 1% uranyl acetate in Maleate buffer for 60 min, washed with maleate buffer and dehydrated by washing with increasing concentrations of cold ethanol from 30% through 95% once each time for 5 min followed by 100% ethanol three times for 10 min each at room temperature. The tissue was incubated in propylene oxide for 15 min each for three times and propylene oxide was removed by overnight incubation with 1:1 propylene oxide/eponate 12 at room temperature followed by pure eponate 12 medium for 4–6 h. The tissue sample was polymerized and semi-thin section of 1 µM were cut with diamond knife stained with toluidine blue for observation in a Leica DM5500 light microscope. Ultra-thin sections of 85 nm were cut with diamond knife, stained with uranyl acetate and lead citrate, and observed with a Tecnai G2 SpiritBT, electron microscope operated at 60 kV.

### Statistics

Data are expressed as mean ± SEM. Statistical comparisons were performed using an unpaired Student’s *t*-test for two samples comparison and analysis of variance (ANOVA) was performed for multiple comparisons of paired echocardiography parameters and for the qRT-PCR analysis. Post-hoc analysis was performed with a Scheffe test. A value of *p < 0.05 was considered significant.

### Ethics approval

All the animal studies were approved by the Cleveland Clinic IACUC.

## Supplementary Information

Supplementary Information 1.

Supplementary Information 2.

## Data Availability

Primary data including global cardiac proteomics data is available and the authors will comply with reasonable requests.

## Abbreviations

EGFR: Epidermal growth factor receptor
ERBB2: Erythroblastic oncogene B
Erbb2: Proto-oncogene Neu
TAC: Transverse aortic constriction
DCM: Dilated Cardiomyopathy
miRNA-7: MicroRNA-7
