# Supplementary material for: Cardiac expression of microRNA-7 is associated with adverse cardiac remodeling
Source: Sci Rep. 2021 Nov 10;11:22018. doi: 10.1038/s41598-021-00778-6 (PMC8581024; doi:10.1038/s41598-021-00778-6)
Supplement: Supplementary file 1 — Supplementary Information 1. [file 41598_2021_778_MOESM1_ESM.pdf]

Supplementary Figure 1

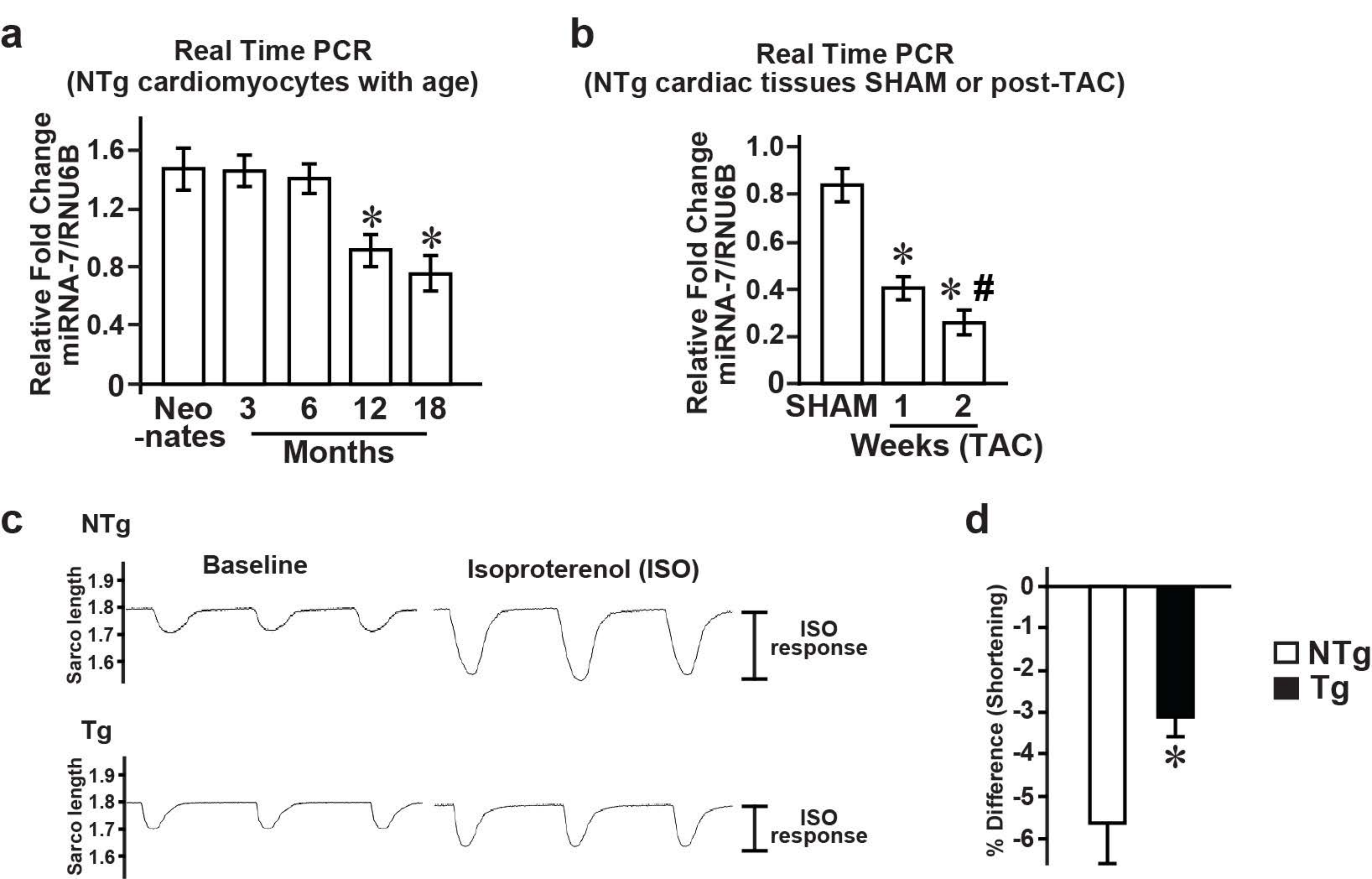

GO: Molecular function

Supplementary Figure 2

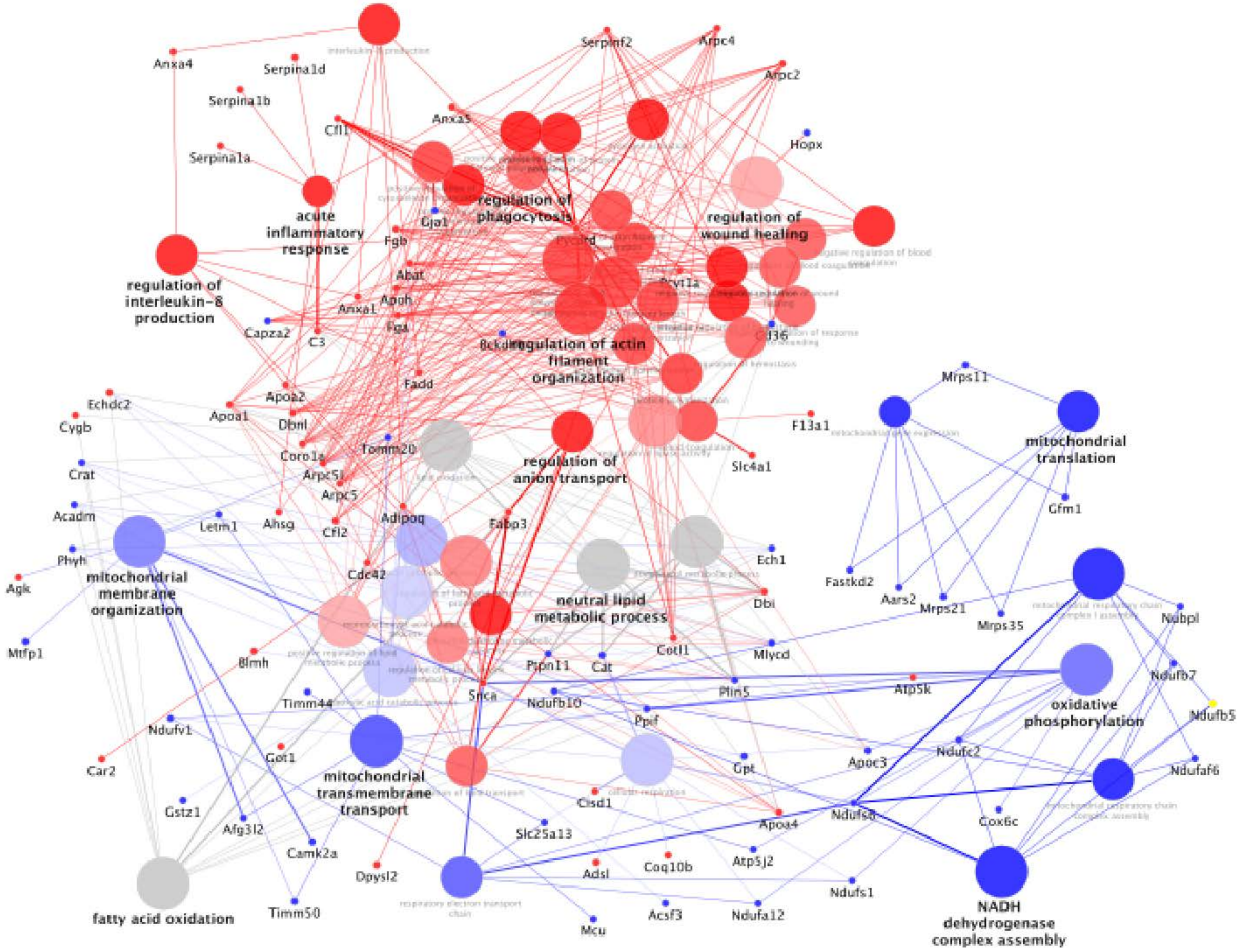

GO: Cellular component

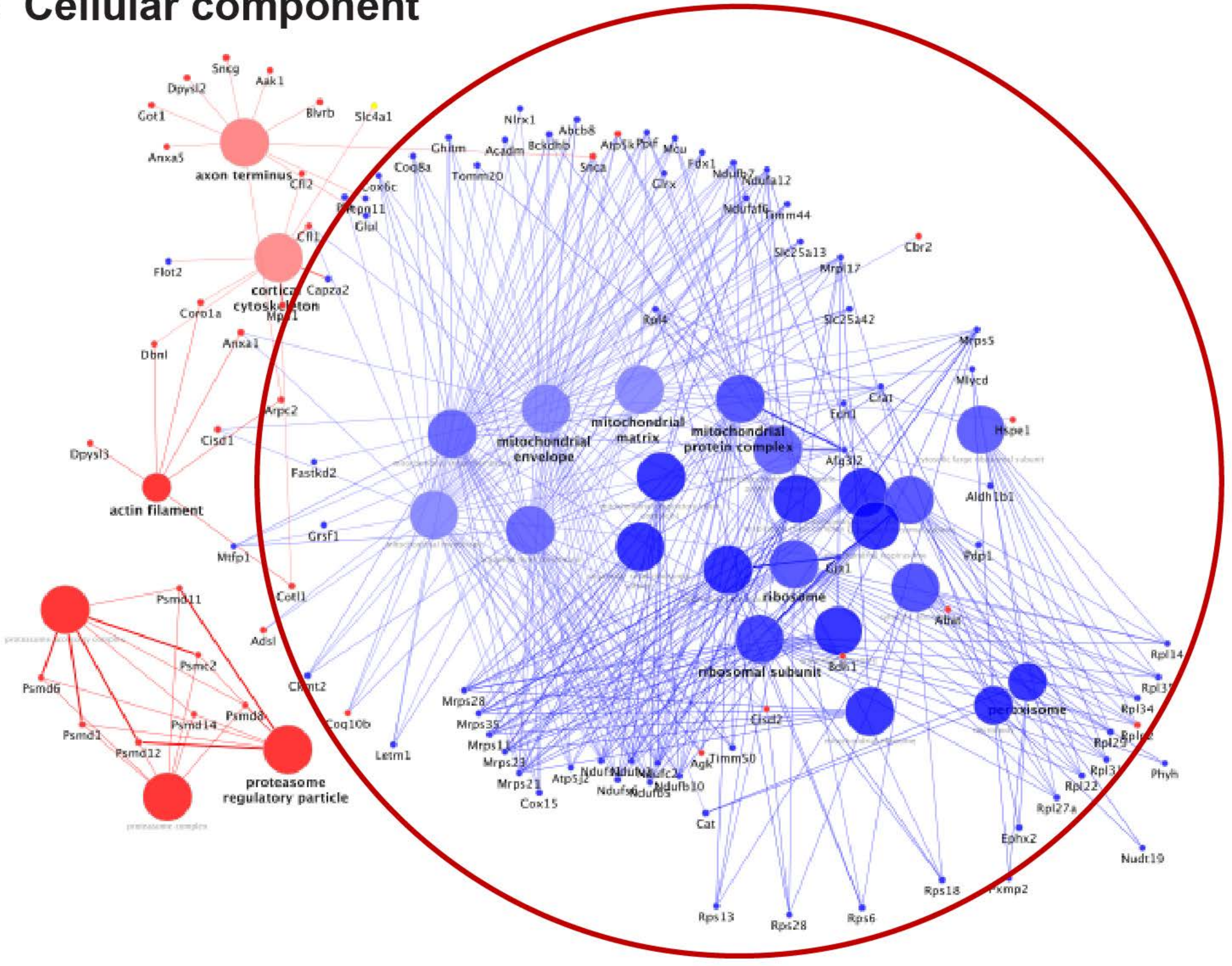

Supplementary Table 1: Comparison of proteins altered in the cardiac proteomic study (miRNA-7 Tg over NTg) to the miRNA-7 predicted targets in TargetScan and miRDB prediction database (Blue coded proteins - Downregulated; Red coded proteins – Upregulated)

A: Proteins identified to be common in all three analysis (proteomics, TargetScan and miRDB)

Seven proteins/genes were identified (all downregulated in miRNA-7 Tg heart)

1) Snca - α-synuclein
2) Ghitm - Growth hormone hormone-inducible transmembrane protein
3) Nlrx1 - NOD-like receptor family member X1
4) Parp1 - poly(ADP-ribose)polymerase-1
5) Vps26a - Vacuolar protein sorting-associated protein 26
6) Ppif - Cylophilin D
7) Plec - Plectin

B: Proteins identified to be common in all two analysis (proteomics and TargetScan)

Six proteins/genes were identified (that are upregulate or downregulated in miRNA-7 Tg heart)

1) Aak1 - AP2-associated protein kinase 1
2) Ablim1 - Actin Binding LIM Protein 1
3) Bzw1 - Basic leucine zipper and W2 domain-containing protein 1
4) Col1a2 - Collagen, Type I, Alpha 2
5) ERBB2 - Erythroblastic oncogene B
6) Timm20 - Translocase Of Inner Mitochondrial Membrane 20

B: Proteins identified to be common in all two analysis (proteomics and miRDB)

Five proteins/genes were identified (that are upregulated or downregulated in miRNA-7 Tg heart)

1) Aars2 - Alanyl—tRNA synthetase 2, mitochondrial,
2) Bcap31 - B Cell Receptor Associated Protein 31
3) Cat - Catalase
4) Col6a1 - Collagen, Type 6, Alpha 1
5) Gja1 - Gap junction alpha-1 protein - Connexin 43

Supplementary Fig 4

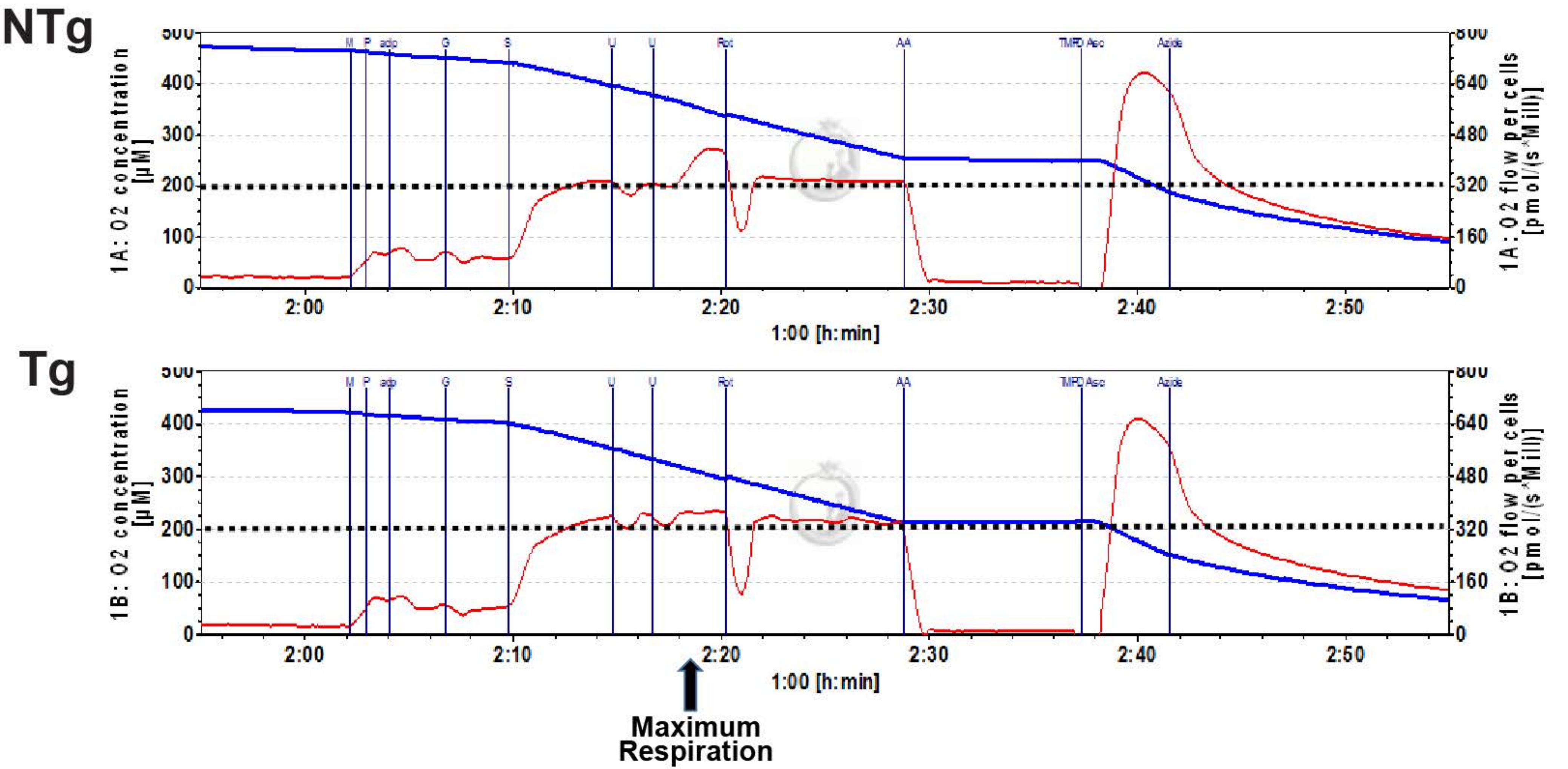

Supplementary Fig 5

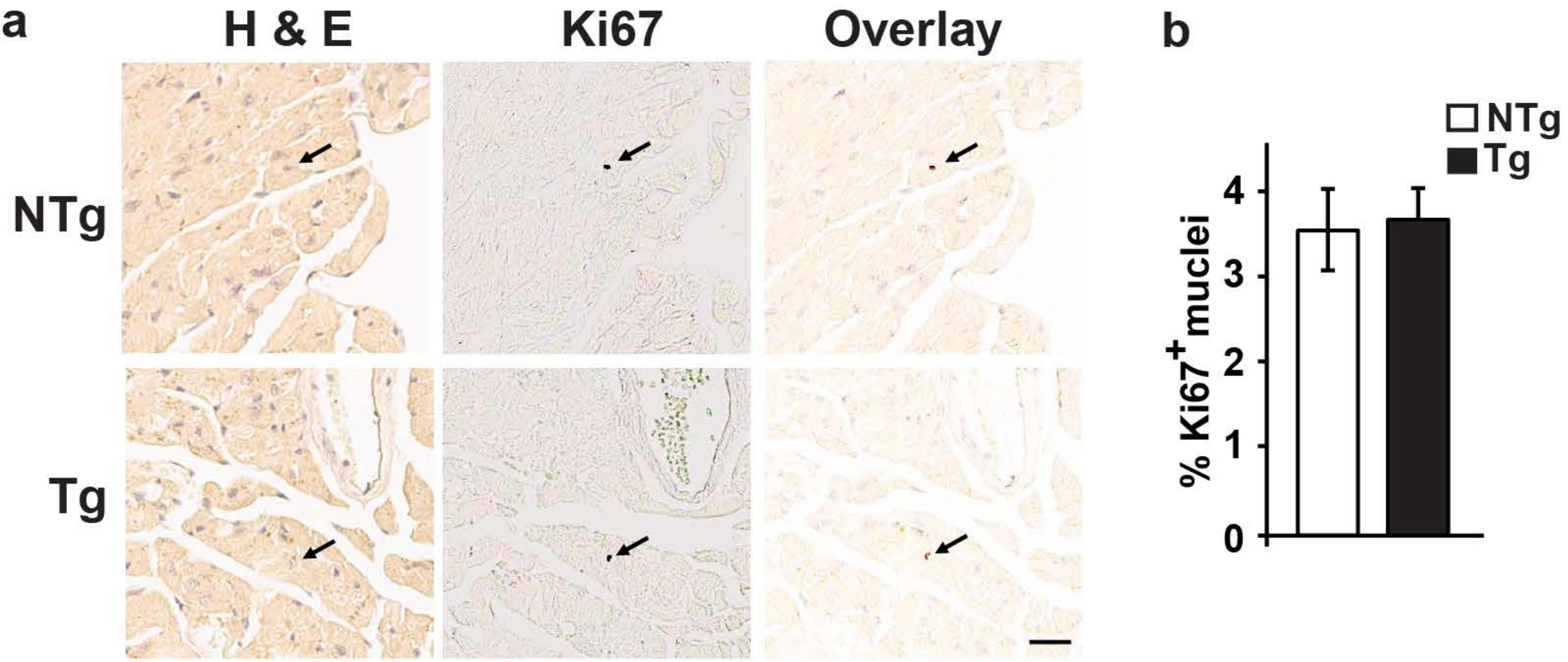

Supplementary Figure Legends

Supplementary Figure 1. **a**, Cardiomyocytes were isolated from neonatal mice or adult cardiomyocytes were isolated from 3, 6, 12 or 18 months old NTg and miRNA-7 Tg mice. mRNA was isolated from these cardiomyocytes and qRT-PCR was performed to evaluate changes in miRNA-7 expression. While no appreciable changes were observed in the early ages (neonates, 3 or 6 months), there was a significant reduction in miRNA-7 expression at 12 and 18 months of age indicating an age-based reduction in expression of miRNA-7 suggesting a role in age-based cardiac responses. *$p<0.005$ vs. neonates, 3 or 6 months (n=6). **b**, qRT-PCR analysis on the cardiac tissues performed following SHAM or TAC for 1 or 2 weeks. The normalized qRT-PCR values are presented in Fig. 3b, while here the relative values to RNU6B is presented to provide insight on the expression of miRNA-7 in NTg. *$p<0.001$ vs. SHAM; #$p<0.01$ vs. 1 week TAC (n=8). **c & d**, Adult cardiomyocytes were isolated from 3 months old NTg and Tg mice and were paced at 1 Hz (using the IonOptix Myopace system) to record the baseline cardiomyocyte contraction. Following steady baseline contractions, the cells were stimulated at 10 μM isoproterenol (ISO) and cardiomyocyte contractions were continuously recorded using IonOptix System. **c**, Baseline myocyte contractility in NTg (upper left panel) and in vitro ISO-stimulated myocyte contractility (upper right panel). Baseline contractility in Tg (lower left panel) and in vitro ISO-stimulated myocyte contractility (lower right panel). **d**, % change in sarcomere length in response to in vitro ISO stimulation. *$p<0.005$ vs. NTg (n=8 mice each of miRNA-7 Tg or NTg; 15-20 adult cardiomyocytes from each NTg or TG mice).

Supplementary Figure 2. Gene ontology (GO) molecular functional networking analysis representing the higher magnification iteration of Figure 5b.

Supplementary Figure 3. Gene ontology (GO) cellular component analysis representing the higher magnification iteration of Figure 5c.

Supplementary Figure 4. *Representative tracing of high-resolution respirometry to quantify respiration of mitochondria isolated from adult cardiomyocytes* – Mitochondria were isolated from cardiomyocytes from adult mice and placed in the oxygraph chamber in MirO5 buffer.

After initial stabilization, 2 mM malate and 2.5 mM pyruvate were added followed sequentially by 2.5 mM ADP; 10 mM glutamate; 10 mM succinate. Subsequently, FCCP (U) an uncoupler of oxidation and phosphorylation was added increments of 0.5µM to measure maximum respiration. This was followed by 375 nM rotenone (R) to inhibit complex I of electron transport chain (ETC) to measure rotenone sensitive and insensitive respiration. Then 2.5 µM antimycin A (Aa) (complex III inhibitor) was added to determine non-mitochondrial respiration followed by 2 mM ascorbate (Asc) and 2 mM tetramethyl p-phenylene diamine (TMPD) to test complex IV activity and finally, 50 mM sodium azide (Az) to inhibit complex IV activity. The black-dotted line provides a reference for the maximum respiratory rate in the NTg compared to the miRNA-7 Tg mitochondria that shows reduced maximum respiratory capacity despite similar levels of oxidative phosphorylation rates. All data expressed as mean±SD from at least 3 biological replicates from cardiomyocytes. $*P < 0.05$; compared to respective controls.

Supplementary Figure 5. *Ki67 immunohistochemistry* – Formalin-fixed paraffin embedded sections were deparafinized in xylene and hydrated through a series of washes in decreasing concentration of alcohol to sterile water[1,2]. Antigen retrieval was performed with 10 mM sodium citrate (pH 6.0) for 10 minutes in incubator at 95ºC and removed and placed at room temperature to cool. After the slides attained room temperature the sections were washed several times with sterile water and the endogenous peroxidase was blocked by using 3% H2O2 in 1 X TN buffer (150 mM, NaCl, 100 mM Tris-HCl (pH 7.5) for 20 minutes at room temperature followed by several washes with 1 X TN buffer. The slides were then incubated with anti-Ki67 antibody (Abcam, ab16667; 1:200) followed by the standard DAB staining. In parallel, the next subjacent section was stained with H & E for and DAPI for evaluating the nuclei. The sections were imaged using the slide scanner-Aperio AT2 (Leica Biosystems) and analysis for the positive nuclei was performed using the Image-Pro 10 (Media Cybernetics). **a**, Heart sections from NTg (upper panel) and Tg (lower panel) were stained with H & E or anti-Ki67 antibody. Scale bar 200 µm (n=4). **b**, Quantitation of myocardial $Ki67^+$ over nuclei stained with DAPI and assessment showed no appreciable differences between the NTg and miRNA-7 Tg.

References:

1 Eminaga, S., Teekakirikul, P., Seidman, C. E. & Seidman, J. G. Detection of Cell Proliferation Markers by Immunofluorescence Staining and Microscopy Imaging in

Paraffin-Embedded Tissue Sections. *Curr Protoc Mol Biol* **115**, 14 25 11-14 25 14, doi:10.1002/cpmb.13 (2016).
2 Gude, N. *et al.* Akt promotes increased cardiomyocyte cycling and expansion of the cardiac progenitor cell population. *Circ Res* **99**, 381-388, doi:10.1161/01.RES.0000236754.21499.1c (2006).
