# Supplementary figures and images for: Cardiac expression of microRNA-7 is associated with adverse cardiac remodeling
Source: Sci Rep. 2021 Nov 10;11:22018. doi: 10.1038/s41598-021-00778-6 (PMC8581024; doi:10.1038/s41598-021-00778-6)

# Figure 2C

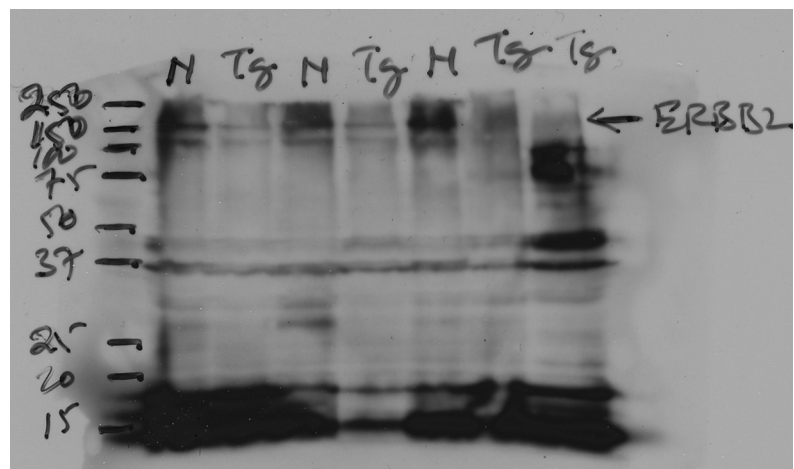

# Figure 2D

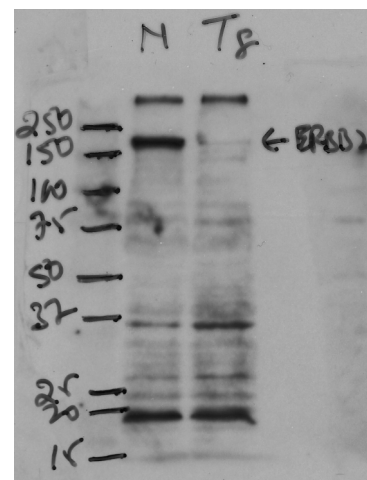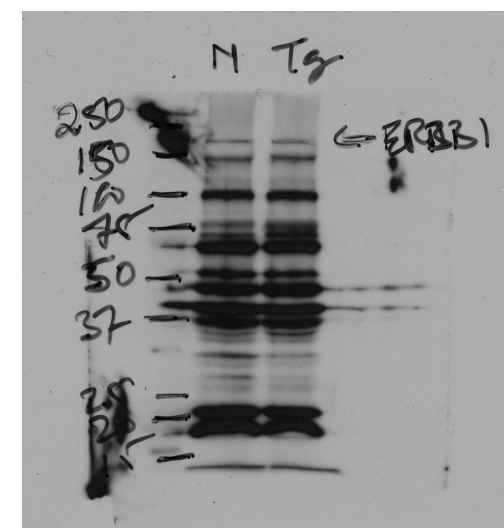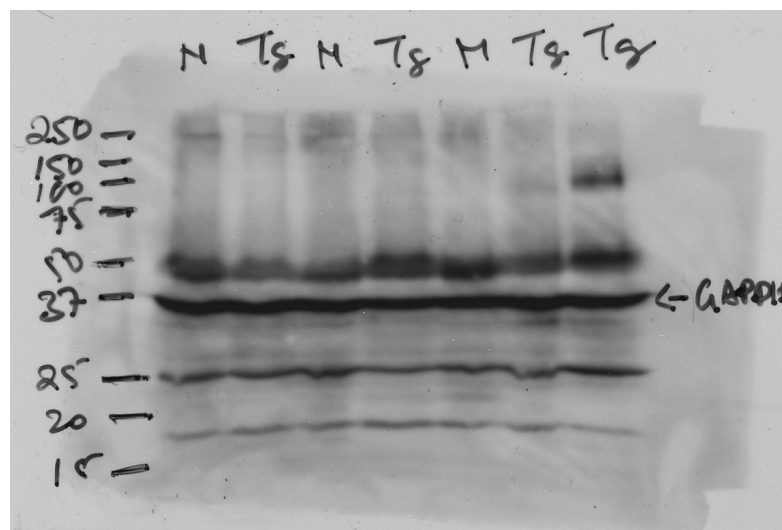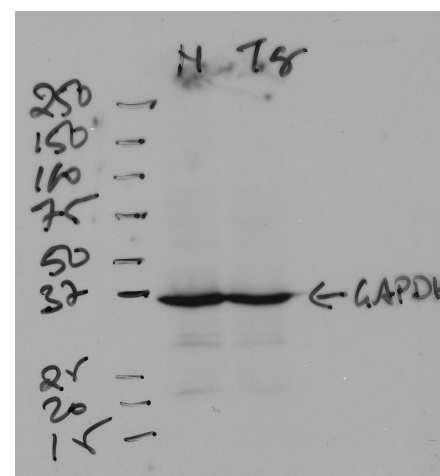

Figure 6a

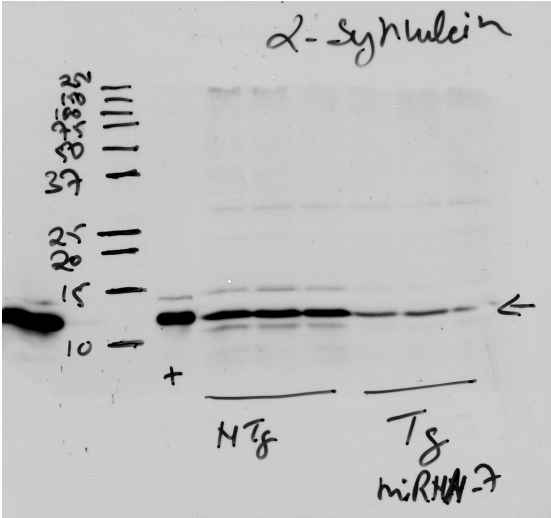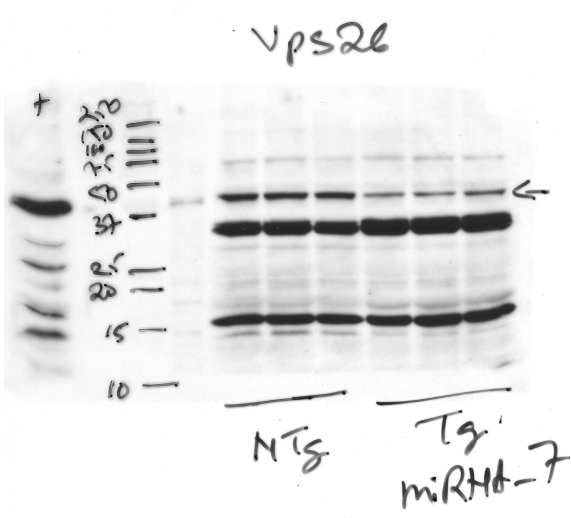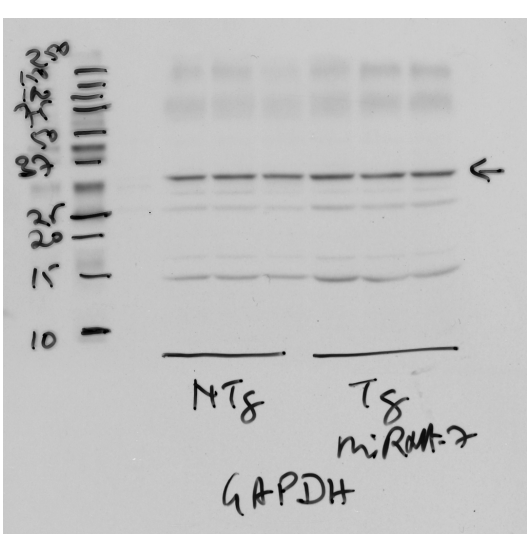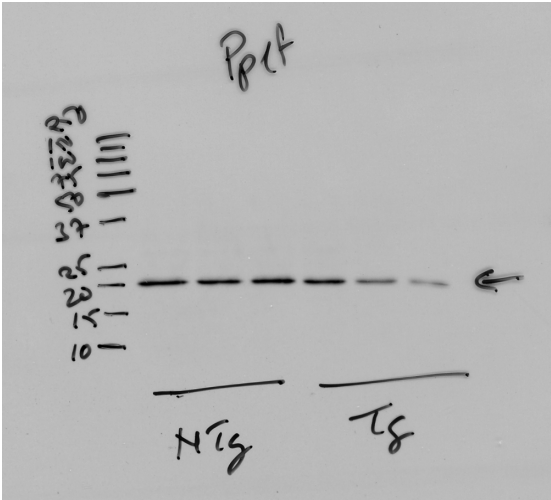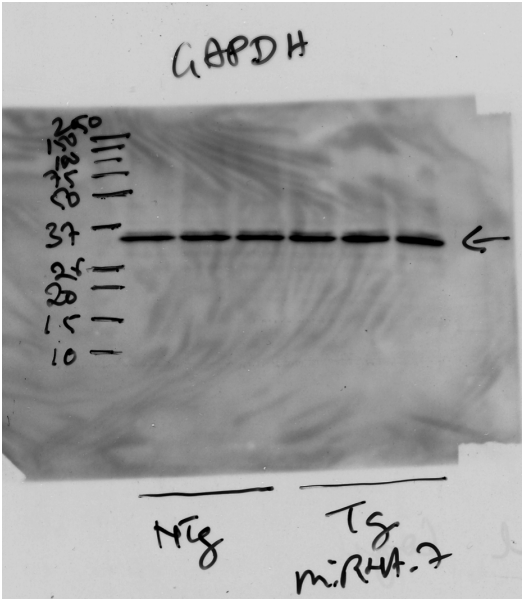

Figure 6b

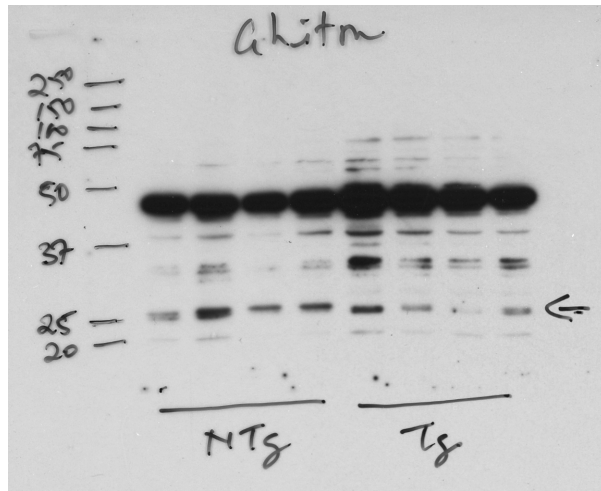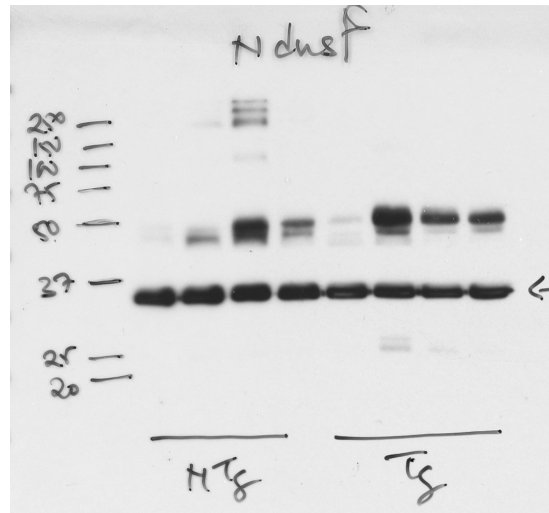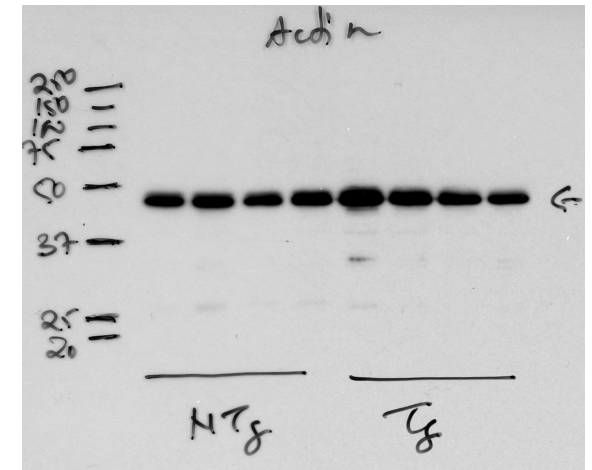

Supplement: Supplementary file 2 — Supplementary Information 2. [file 41598_2021_778_MOESM2_ESM.pdf]
